# Targeting stiffness-dependent YAP/TAZ restores angiogenesis dynamics impaired by ALK1 knockout *in silico*

**DOI:** 10.1371/journal.pcbi.1013561

**Published:** 2026-07-16

**Authors:** Margot Passier, Sandra Loerakker, Tommaso Ristori

**Affiliations:** 1 Department of Biomedical Engineering, Eindhoven University of Technology, Eindhoven, the Netherlands; 2 Institute for Complex Molecular Systems (ICMS), Eindhoven University of Technology, Eindhoven, the Netherlands; University of Connecticut School of Medicine, UNITED STATES OF AMERICA

## Abstract

Hereditary Hemorrhagic Telangiectasia (HHT) is a currently incurable genetic disorder caused by loss-of-function mutations in the ALK1-BMP9 pathway, leading to dysregulated angiogenesis and consequential vascular malformations. Recent experiments also implicate the mechanotransducers YAP/TAZ in HHT pathology. However, how YAP/TAZ stiffness sensitivity and signaling activity contribute to aberrant HHT angiogenesis remains poorly understood. Here, we extended our previous computational framework of stiffness-mediated YAP/TAZ-VEGF-NOTCH crosstalk to account for ALK1 signalling and predict the resulting angiogenic temporal dynamics. Our simulations predicted that ALK1 knockout impairs NOTCH activation, slowing endothelial phenotypic selection and shuffling while enhancing filopodia activity, features corresponding with hypersprouting. These effects were most pronounced in low stiffness environments, consistent with the previously observed prevalence of HHT vascular malformations in low stiffness organs. Importantly, the temporal dynamics of endothelial phenotypic selection and shuffling, as well as key protein activity levels, were partially restored by direct or cytoskeleton-mediated inhibition of YAP/TAZ resulting from increased NOTCH activation. These computational findings offer more mechanistic insight into the signalling pathways and temporal dynamics of endothelial phenotypic selection underlying HHT vascular anomalies, and suggest that targeting YAP/TAZ and endothelial stiffness sensitivity may offer a promising therapeutic strategy to restore physiological angiogenesis.

## Author summary

Mechanical cues such as extracellular matrix stiffness, sensed by endothelial cells lining blood vessels, play a critical role in angiogenesis, the process of blood vessel formation from pre-existing vessels. Understanding the impact of these cues on angiogenesis in health and in diseased conditions could help guide new treatments for angiogenesis-related disorders, such as Hereditary Hemorrhagic Telangiectasia (HHT). In HHT, genetic mutations disrupt normal vessel development, leading to malformations that can rupture and have detrimental consequences. Here, we developed a computational model to investigate the effects of HHT-associated genetic mutations on angiogenic signaling of endothelial cells exposed to different stiffnesses. Simulations predicted that the mutation impairs endothelial phenotypic selection and shuffling, necessary for physiological angiogenesis. The mutation effects were the largest in soft organs and the simulations showed mechanotransducers YAP/TAZ as possible targets to restore physiological signaling. Therefore, with this model, we have created a platform to simulate endothelial cell behavior in HHT patients, allowing us to explore mechanosensitive pathways as potential targets potentially extending future treatment options.

## Introduction

Hereditary hemorrhagic telangiectasia (HHT) is a currently incurable genetic disease, affecting approximately 1 in 5000 people, characterized by the development of vascular malformations [1–3]. The most commonly observed malformations are telangiectasia, small widened vessels in the mucosa and superficial skin layers [2,4]. These fragile vessels are prone to rupture, often resulting in recurrent bleeding and anemia [5]. Moreover, patients frequently develop arteriovenous malformations (AVMs), direct connections (shunts) between arteries and veins replacing the physiological capillary bed [2,6,7]. Such AVMs predominantly form in the lungs, liver, and brain [2,4,8], potentially resulting in serious complications such as rupture and consequential bleeding or organ failure. In most cases (over 92%), HHT is caused by mutations affecting the activin-receptor like kinase 1 (ALK1) – Bone Morphogenic Protein 9 (BMP9) signaling pathway [2,9–14]. Increasing evidence shows that these mutations dysregulate angiogenesis, the process of new blood vessel formation from pre-existing vessels [15], thereby leading to the vascular malformations mentioned above. However, the underlying mechanisms are still unclear, hindering the development of definitive cures.

Under physiological conditions, ALK1-BMP9 signaling regulates Vascular Endothelial Growth Factor (VEGF)-NOTCH signaling crosstalk, a key regulator of sprouting angiogenesis [16]. Briefly, VEGF kickstarts sprouting angiogenesis by activation of the VEGF-Receptor 2 (VEGFR2), which induces previously quiescent endothelial cells (ECs) to adopt the tip and stalk phenotypes. Specifically, VEGFR2 activation upregulates filopodia formation and expression of the NOTCH ligand Delta-like ligand 4 (DLL4), thereby inducing a migratory tip phenotype. Simultaneously, DLL4 binds to and activates the NOTCH1 receptor on neighboring cells, causing downregulation of VEGFR2 and upregulation of VEGF-Receptor 1 (VEGFR1, a decoy receptor for VEGF [17–19]). The VEGF-sensing ability is therefore downregulated in these NOTCH1-activated cells and they are induced to acquire a stalk cell phenotype [20,21]. This process is highly dynamic, as cells continue to rearrange their position and to switch phenotype [22,23]. Knockout (KO) of the BMP9-receptor ALK1 (ALK1 KO), the causal mutation of the HHT genetic subtype HHT2, disrupts this balance of angiogenic stimuli [24]. It leads to decreased activity of NOTCH target genes HES and HEY, and consequently downregulated VEGFR1 activity and upregulated VEGFR2 activity [25]. This causes overactivity of pro-angiogenic stimuli through active VEGFR2, which is thought to be one of the underlying mechanisms causing the vascular anomalies observed in HHT2 patients.

In addition to directly affecting NOTCH targets, ALK1 activation has recently been shown to upregulate the expression of Lunatic Fringe (LFNG), a glycosyltransferase that enhances NOTCH1-DLL4 binding affinity and activation [26–28]. Al Tabosh et al. identified LFNG as one of the few genes consistently dysregulated in newborn HHT patients carrying the ALK1 heterozygous mutation [26]. This suggests a central role of LFNG in HHT and its corresponding aberrant angiogenesis. In support of this, LFNG inhibition has been shown to cause hypersprouting [27]. Furthermore, Ristori et al. have recently shown that BMP9 regulates NOTCH1 and sprouting angiogenesis by increasing the expression of LFNG [29]. Hence, these studies implicate LFNG as one of the key players in HHT2.

Another recent study has shown that mechanotransducers Yes-Associated Protein (YAP) and transcriptional coactivator with PDZ-binding motif (TAZ) also play a major role in HHT2 [2,6]. YAP/TAZ, transcriptional co-activators of the Hippo pathway, are mechanosensors that are mainly located in the cytoplasm for cells on substrates with relatively low stiffness, while their nuclear localization (and thus regulation of gene expression, generally via interaction with the transcriptional enhanced associate domain, TEAD) increases with increasing substrate stiffness [30–34]. Nuclear translocation of YAP/TAZ as a result of mechanical cues is initiated by integrin binding, followed by multiple cytoskeletal processes culminating in stress fiber formation and consequential nuclear deformations allowing YAP/TAZ nuclear translocation [35–37]. YAP/TAZ nuclear translocation is also regulated through the Hippo pathway and angiomotins, which can sequester YAP/TAZ in the cytoplasm resulting from phosphorylation or AMOT (angiomotin proteins) binding [38–40]. VEGF also influences this process; VEGFR2 activation upregulates focal adhesion kinase (FAK) phosphorylation, involved in the stress fiber formation and nuclear translocation of YAP/TAZ [41,42]. Interestingly, inhibition of YAP/TAZ has recently been shown to prevent AVM formation in ALK1 KO mice via rescued EC polarization sensitive to blood flow [6], indicating that YAP/TAZ are involved in HHT AVMs. However, how YAP/TAZ sensitivity to stiffness and its crosstalk with angiogenic signaling pathways fit into this picture are currently unclear.

Previous experiments have shown that YAP/TAZ downregulate the expression of LFNG and DLL4 [43,44]. By using computational simulations, we have recently shown that this YAP/TAZ-NOTCH1 crosstalk could explain the angiogenic response to stiffness [45]. In particular, we found that, through these interactions, YAP/TAZ is a temporal regulator of EC phenotypic selection speed [45] and thus vascular topology [46,47]. In the present study, we hypothesized that this crosstalk might be essential in HHT2 as well. More specifically, we computationally investigated whether the YAP/TAZ-sensitivity and interaction with the NOTCH pathway can explain and potentially be targeted to rescue the abnormal EC behavior and phenotypic selection observed in response to ALK1 KO [29,48], specifically in tissues prone to AVM formation in HHT2, such as the liver, characterized by relatively low stiffness [1,3,24,49–54].

In the field of angiogenesis, there have been many advances resulting from computational models addressing key aspects of EC behavior [23,29,46,47,55–68]. An integration of simulations and experiments for example, led to the discovery of EC phenotypic and positional shuffling [22,23], and to the observation that the EC phenotypic selection and shuffling rates influence vascular network topology. For instance, faster patterning dynamics are associated with the formation of denser networks [46]. For mechanisms also involved in HHT specifically, computational approaches have been adopted to establish the importance of LFNG in BMP9-regulated vascular patterning [29], and to explore VEGFR1-VEGFR2 competition, elucidating its impact on sprout directionality [69]. Moreover, computational studies focused on HHT2 mainly addressing the characteristics of mutations and diagnostics, contributed to the emerging view that HHT2 might be substantially underdiagnosed [70].

In this study, we developed a computational model to gain more insight into the role of YAP/TAZ stiffness sensitivity and its effect in HHT2. Specifically, we extended our previous YAP/TAZ-VEGF/NOTCH1 framework for stiffness-regulated tip/stalk pattern formation based on ordinary differential equations (ODEs) [36,45,62] by: (i) including the effects of BMP9-ALK1 signaling on the VEGF-NOTCH1 crosstalk [29]; (ii) differentiating between VEGFR1 and VEGFR2 [17,19,22,71]; and (iii) accounting for VEGF-induced FAK activation [41,42,72,73]. The ensuing simulations, focusing on EC phenotypic selection, allowed us to dive into the effects of YAP/TAZ stiffness sensitivity on ECs that are affected by the ALK1 KO mutation. Our simulations indicated that ALK1 KO significantly altered signaling dynamics, leading to decreased NOTCH activity (especially on lower stiffnesses), causing decreased lateral inhibition, thereby slowing down phenotypic selection. Furthermore, we found that ALK1 KO ECs have an impaired ability to switch phenotypes and studied downregulation of cytoskeletal elements as possible new targets in HHT2. We found that YAP/TAZ inhibition could partially rescue physiological behavior, such as phenotypic selection time, but not completely, potentially because LFNG expression was not completely restored. We highlight the potential of the identified signaling network and developed computational model as a screening and simulation tool for conducting more *in silico* experiments.

## Results

### The extended model captures VEGF- and stiffness-dependent YAP/TAZ nuclear translocation and tip/stalk temporal dynamics

**Fig 1:**
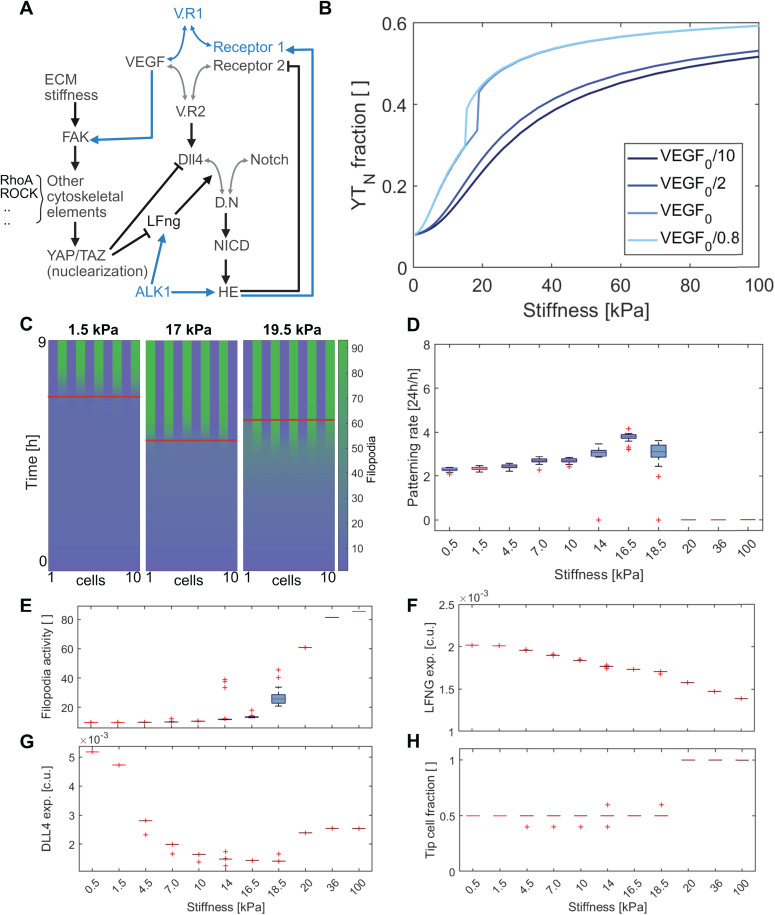
The extended model captures the stiffness- and VEGF-dependent regulation of YAP/TAZ, and the biphasic response of tip-stalk selection to stiffness. Overview of the model main signaling components, with the added elements highlighted in blue **(A)**. Mean trends of YAP/TAZ nuclear fractions for different amounts of VEGF **(B)** for the VEGF concentration used to instigate patterning VEGF_0_. Representative images of filopodia activity over time, for three different stiffnesses (1.5, 17 and 19.5 kPa). The red lines indicate the timepoint at which the pattern is considered established **(C)**. The patterning rate (24h/patterning time) **(D)**, filopodia activity prior to pattern formation **(E)**, expression of LFNG **(F)**, expression of DLL4 **(G)** and fraction of tip cells (H) are shown. All simulations were repeated 25 times, with the boxes in B-F depicting quartiles 2 and 3, the red lines indicating the medians, the whiskers indicating the min- and maxima and the red plusses indicating the outliers. For **(B)**, only the mean is plotted as the standard deviation was omitted because it was extremely small and visually not discernible. Created in BioRender. Passier, **M.** (2026) https://biorender.com/esq9brp.

Fig 1A shows a schematic of the computational model, with model extensions compared to previous versions [36,45,62] highlighted in blue. Briefly, the original YAP/TAZ model assumes that increasing ECM stiffness induces phosphorylation of FAK, with consequently increased activity of cytoskeletal elements culminating in augmented actomyosin contractility and subsequent YAP/TAZ nuclear translocation [36,45]. Nuclear YAP/TAZ are assumed to downregulate the expression of LFNG and DLL4 [43,45], thereby influencing the VEGF-NOTCH crosstalk which features two feedback loops [62]: a positive feedback loop in which VEGFR2 activation leads to filopodia formation and extension with consequently higher potential to sense VEGF [60]; and a negative feedback loop where VEGF upregulates DLL4 expression, inducing NOTCH activation in neighboring cells that causes downregulation of their VEGFR2 expression [74]. Here, we extended the framework by assuming that: (i) active VEGFR2 induces FAK activation in synergy with stiffness [75–78]; (ii) HE upregulates the expression of VEGFR1 [22], a decoy VEGF receptor; and (iii) ALK1 enhances HE and LFNG expression [26,29]. We first verified whether our computational model, extended with the VEGF-FAK interaction and VEGFR1–2 distinction, could reproduce the experimentally-observed YAP/TAZ nuclear fractions dependent on VEGF activity and stiffness [41,79]. Similar to the original model [36,45], the extended model predicted that YAP/TAZ nuclear translocation increased in response to stiffness following a logistic shape (Fig 1B, blue line with reference parameter VEGF_0_). This resulted from the stiffness-mediated increase in phosphorylated FAK. Due to the inclusion of VEGF-mediated upregulation of phosphorylated FAK in the model, the simulations could also capture the increased nuclear fraction of YAP/TAZ caused by increased VEGF exposure (Fig 1B). Next, we verified that the model could capture the trends in angiogenic patterning behavior in response to stiffness observed in our previous computational study [45]. Consistent with our previous simulations [45], the extended model predicted a salt-and-pepper pattern in terms of filopodia activity (determined via Eq. S7), with filopodia-rich cells alternated with cells exhibiting few filopodia (Fig 1C for representative simulations). This is in line with the tip-stalk pattern characterizing physiological angiogenesis [80–82]. The rate at which this pattern formed (see Methods for the pattern rate quantification) depended on stiffness (Fig 1C and 1D): the simulations captured the biphasic relationship between patterning rate and stiffness, predicting the highest patterning rate for an intermediate stiffness (significantly different from the patterning rates at 14 and 18.5 kPa, with p < 0.00001) [83,84] (Fig 1C and 1D), as well as increased filopodia activity for stiffer substrates (Fig 1E), in agreement with our previous simulations [45]. More in detail, on stiffnesses below the optimum (16.5 Kpa in our simulations, Fig 1C and 1D), cells were relatively slow to form a tip-stalk pattern and showed little filopodia activity prior to this (Fig 1C and 1E). At 16.5 kPa, ECs achieved the fastest tip-stalk pattern formation (Fig 1C and 1D). Above this threshold, tip-stalk pattern formation slowed down, with ECs exhibiting relatively high filopodia activity prior to patterning, indicative of hypersprouting (Fig 1C-1E). The increased nuclear YAP/TAZ content upon increased stiffness (Fig 1B), led to more DLL4 and LFNG inhibition (equations S8, S12-13, Fig 1F and 1G). DLL4 expression was however predicted to recover for stiffnesses above 20 kPa (Fig 1G), as also observed in our previous study [45]. This resulted from the YAP/TAZ-mediated decrease in LFNG, causing impaired NOTCH1 activation and therefore VEGFR2 upregulation, causing all cells to become tip cells (Fig 1H) and express more DLL4 (Fig 1G). These results show that the extended model can predict not only the effects of stiffness on YAP/TAZ and NOTCH1-mediated tip/stalk patterning, but also the effects of VEGF on YAP/TAZ.

### YAP/TAZ inhibition can partially rescue phenotypic selection behavior in ALK1 KO ECs

Next, we investigated the effects of ALK1 KO and YAP/TAZ KO on the temporal dynamics of tip/stalk patterning. Liver stiffness (approximately 5 kPa [50,51,85]) was taken as a reference, given the frequent occurrence of hepatic AVMs in HHT2 [1,3,24]. Consistent with Park et al. [6], we considered three groups: wild-type ECs, ECs with ALK1 KO (implicitly modelled through their effect on HES/HEY and LFNG, Fig 7), and ECs with both ALK1 KO as well as YAP/TAZ KO.

**Fig 2:**
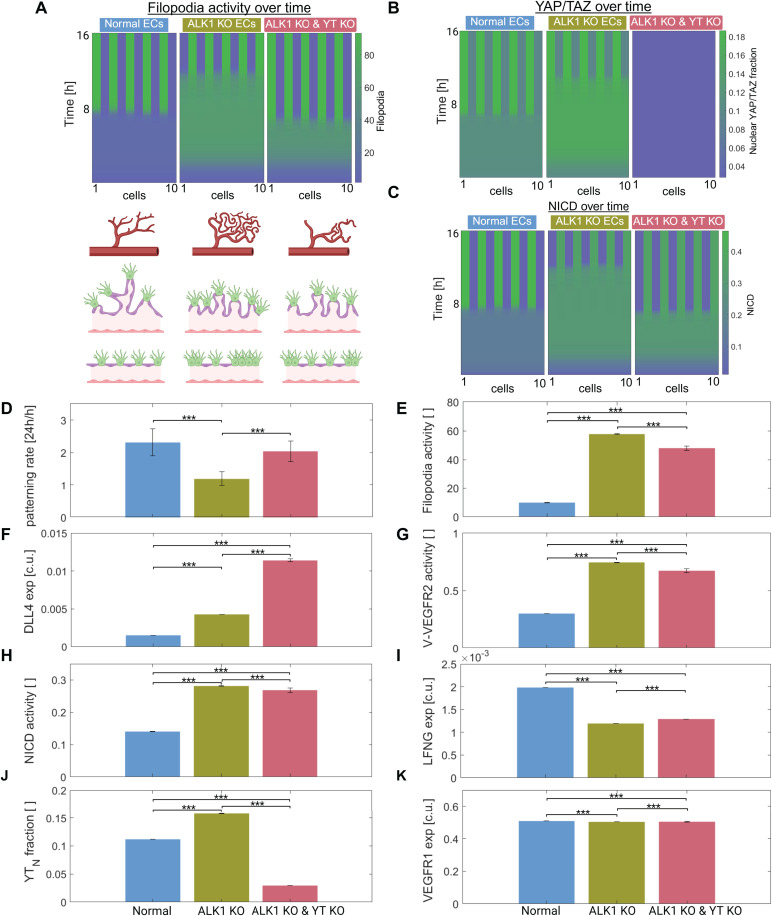
Simulations of ALK1 KO ECs indicate a decreased patterning rate and increased filopodia activity, indicative of hypersprouting. Panels A-C show time courses of filopodia activity **(A)**, YAP/TAZ nuclear fractions **(B)** and NOTCH activity (NICD) **(C)** during the first 16 hours after being provided with a VEGF stimulus to instigate patterning. (A) provides an additional schematic of the predicted vascular topology, indicative of optimal patterning with physiological sprouting (left), hypersprouting (middle), and an in-between case (right). ALK1 KO ECs exposed to liver stiffness (yellow bars) show a decreased patterning rate **(D)**, LFNG expression **(I)**, and minimal decrease in VEGFR1 expression **(K)**, while filopodia activity **(E)**, DLL4 expression **(F)**, VEGFR2 activity **(G)**, NICD **(H)** and YAP/TAZ nuclear fraction **(J)** all increased. Except for DLL4 **(F)**, all behaviors partially returned to wild-type signaling levels (light blue bars) upon YAP/TAZ inhibition (pink bars). All simulations were repeated 25 times, showing the mean with standard deviation represented by the error bars **(D-K)**. To test whether differences between simulated groups differed significantly, we first conducted a one-way ANOVA test, after which we performed the Tukey’s honestly significant test, the results of which are represented by the bars with * above them, *** representing p < 0.001. For E-K, mean activity and expression were determined by averaging the amounts over the timepoints prior to pattern formation. The schematic in (A) was created in BioRender. Passier, **M.** (2026) https://biorender.com/sc3ioao.

Phenotypic selection slowed down significantly upon ALK1 KO (Fig 2A and 2D). In addition to the ECs needing almost twice as much time to establish the tip/stalk pattern (Fig 2A and 2D), filopodia activity (Fig 2A and 2E) as well as DLL4 expression prior to patterning (Fig 2F) increased significantly, suggesting tip-like behavior, indicative of EC hyperactivity and hypersprouting (Fig 2A) [46,47]. This agrees with experimentally observed behavior of ALK1 KO ECs [48,86]. In the simulations, this resulted from the marked increase in VEGFR2 activity (Fig 2G), consistent with experimental findings [25]. Interestingly, it was not only the activity of tip cell indicators (filopodia and DLL4) that increased, but also NOTCH1 activity (or NICD), indicative of stalk cells, prior to patterning (Fig 2C and 2H). This indicates a reduced cellular ability to perform lateral inhibition. Although average NOTCH1 activity prior to patterning increased upon ALK1 KO (Fig 2C and 2H), NOTCH1 activity in patterned stalk cells appeared to be lower compared to that of wild-type ECs (Fig 2C), at least partially resulting from decreased levels of LFNG leading to decreased DLL4-NOTCH1 binding affinity (Fig 2I). YAP/TAZ nuclear fraction was also seen to increase upon ALK1 KO, in agreement with experimental findings [6], especially prior to pattern formation (Fig 2B and 2J). VEGFR1 expression was not much affected by ALK1 KO (Fig 2K). Overall, the ALK1-KO simulations suggested that EC hypersprouting experimentally observed in ALK1-KO angiogenesis [48] could result from delayed tip-cell inhibition and thus delayed tip-stalk pattern formation accompanied by high EC filopodia activity.

**Fig 3:**
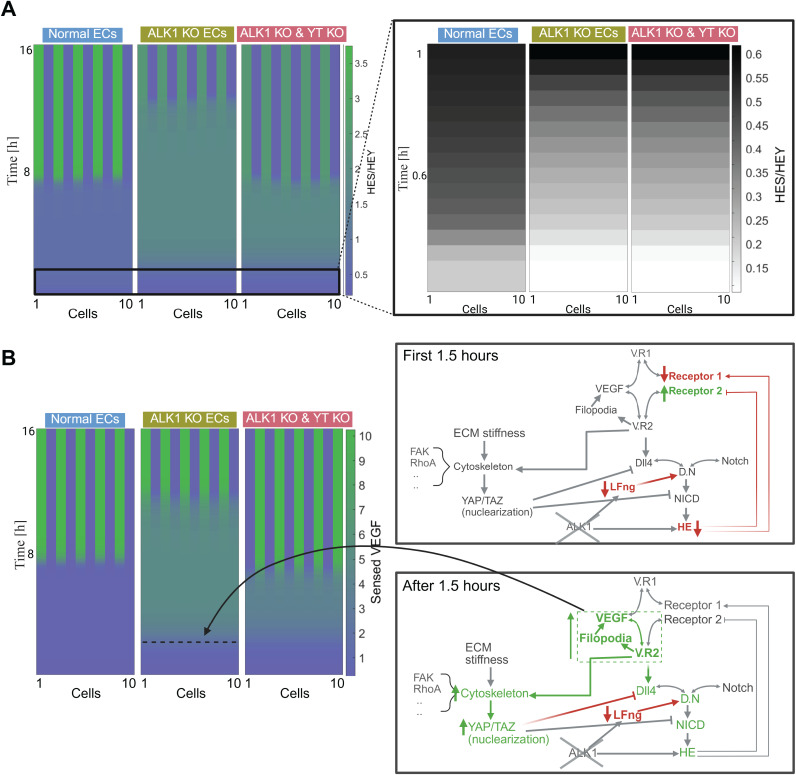
HES/HEY expression initially decreases upon ALK1 KO, but eventually increases due to over-active VEGFR2 signaling. Representative time course of HES/HEY levels for wild-type ECs (left panels, light blue heading), ALK1 KO ECs (middle panels, yellow heading) and ALK1 KO + YAP/TAZ KO ECs (right panels, pink heading) for 16 hours **(A)**. The right window of (A) depicts a zoom-in of the temporal dynamics of HES/HEY expression over the first hour. Time course analysis of VEGF levels, which start to increase after HES/HEY levels of ALK1 KO ECs have increased, resulting from the positive feedback loop consisting of VEGFR2-filopodia-VEGF, depicted in the schematic in the right window **(B)**. The schematic in (B) was created in BioRender. Passier, M. (2026) https://biorender.com/cn5cln3.

For ALK1 KO cells, the expression of NOTCH1 target genes HES/HEY in response to VEGF exposure increased at a slower rate than in wild-type cells (Figs 3A and S1A). After approximately 1 hour, however, HE expression in wild-type cells started to plateau, while HE expression in ALK1 KO cells continued to increase, surpassing that of wild-type cells at 1.25 hours and reaching a plateau after approximately 5 hours. Finally, coinciding with the symmetry breaking events leading to tip-stalk pattern formation (occurring in between 10 and 22 hours), wild-type HE expression increased while the ALK1 KO counterpart decreased, with the latter exhibiting a higher plateau compared to wild type at the end of the simulation, consistent with *in vivo* experiments at 24 hours [29]. This temporal variation in terms of ALK1 KO effects is consistent with previous studies showing that EC responses to ALK1-BMP9, NOTCH and VEGF signaling are temporally varying [29,61,87–89]. In our model simulations, these temporal dynamics of HE expression over time can be explained by the fact that, in wild-type cells, NOTCH-mediated lateral inhibition relatively quickly reduced VEGF sensing and thus rapidly suppressed the VEGF-filopodia-VEGFR2 positive feedback loop, thereby stabilizing DLL4, NICD and HE expression, at low levels (Figs 3 and S1). In contrast, the initially lower expression of HE in ALK1 KO cells led to slower VEGFR2 inhibition and VEGFR1 upregulation, causing VEGFR2 activity to increase over time (S1B Fig). In these ALK1 KO cells, the increased VEGFR2 activity (Fig 2G) led to upregulation of filopodia formation (S1C Fig), more VEGF sensation (Fig 3B) and as a consequence, even more VEGFR2 activity, via the establishment of a positive feedback loop. Increased VEGFR2 activity also upregulated DLL4 expression, leading to increased DLL4-NOTCH1 binding and, finally, increased NICD levels and thus HE expression levels too (Fig 3). The plateaued HE expression in ALK1 KO cells after 5 hours can be explained by HE expression reaching values sufficient to inhibit VEGFR2 and filopodia. For both wild-type and ALK1 KO cells, phenotypic patterning (at 11 and 20 hours respectively) eventually resulted in the establishment of a new plateau (S1 Fig).

Park et al. experimentally demonstrated that inhibition of YAP/TAZ can prevent AVM formation [6]. Consistent with their experiments, we predicted an increase in YAP/TAZ nuclear fraction upon ALK1 KO (Fig 2J). YAP/TAZ inhibition led to a major increase in patterning rate compared to the ALK1 KO case without YAP/TAZ inhibition, bringing it in much closer alignment with wild-type patterning times (Fig 2A and 2D). Upon YAP/TAZ KO, LFNG expression in ALK1 KO ECs increased slightly and DLL4 expression increased two-three fold, therefore leading to stronger lateral inhibition potential (Fig 2F and 2I). Moreover, the marked increase in VEGF levels in ALK1 KO ECs which resulted from the positive VEGFR2-filopodia-VEGF feedback loop, was significantly lower upon YAP/TAZ KO (Fig 3B). Consequently, overall filopodia and VEGFR2 activity prior to pattern formation decreased and cells established the tip/stalk pattern quicker (Fig 2E and 2G). Therefore, despite YAP/TAZ KO not being able to restore all protein levels in the phenotypic selection process, it could nevertheless rescue the tip/stalk patterning rate and thus limit hypersprouting due to increased LFNG and DLL4 expression (Fig 2A). These simulations therefore indicate that YAP/TAZ inhibition may lead to more physiological angiogenesis by mediating the VEGF-NOTCH crosstalk via LFNG and DLL4 regulation.

### YAP/TAZ KO can partially rescue EC phenotypic shuffling impaired by Alk1 KO

**Fig 4:**
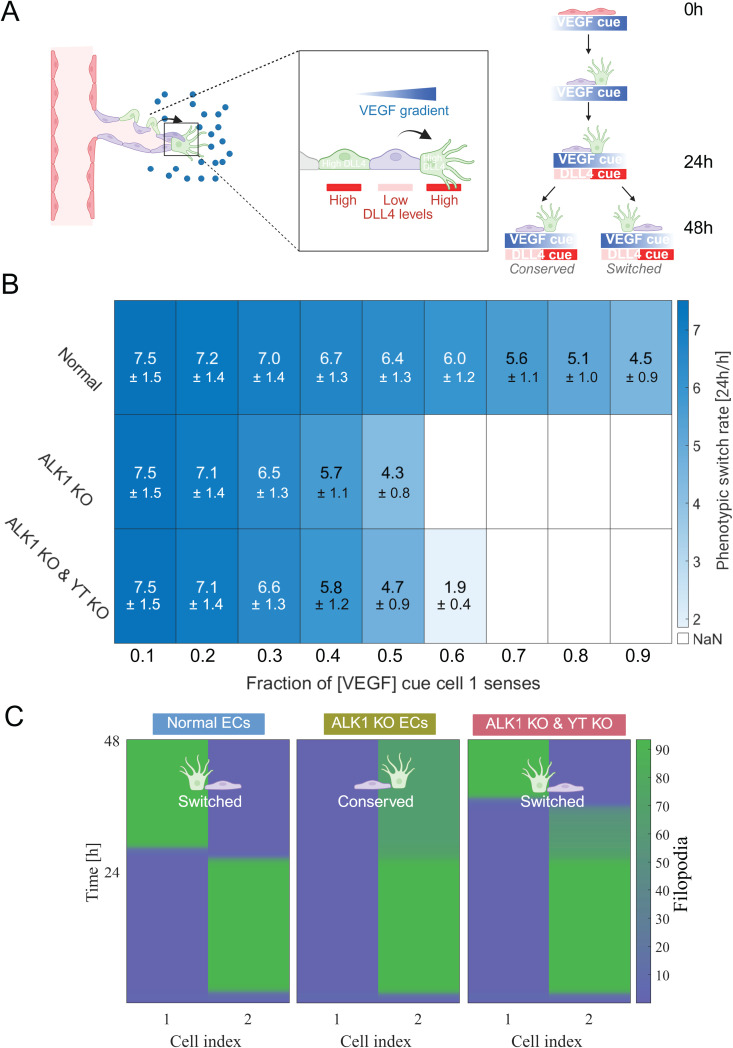
ALK1 KO ECs have impaired phenotypic shuffling ability that can be partially rescued by YAP/TAZ inhibition. Schematic of the different simulated phases mimicking positional switching during angiogenic sprouting **(A)**. A tip and stalk cell switch position, thereby switching the environmental cues that they sense. This is mimicked by first (0-24h) exposing the cells to different VEGF levels, ensuring the right cell becomes a tip cell. After 24h, the environmental cues are switched, mimicking a positional change, leading to either conserved or switched phenotypes. Phenotypic switch rate with standard deviation (simulations conducted 25 times), for the range of different VEGF ratios that was simulated **(B)**. White squares are indicative of conserved phenotypes (i.e., no switch, which are largely present in the ALK1 KO row). Time course analyses of filopodia activity for cell 2 sensing 60% of the amount of VEGF sensed by cell 1, showing switched phenotype for wild-type ECs (left panel), conserved phenotypes for ALK1 KO ECs (middle panel) and delayed switched phenotypes for ALK1 KO & YAP/TAZ KO ECs (right panel) **(C)**. The schematics in (A) and (C) were created in BioRender. Passier, M**.** (2026) https://biorender.com/4gu5kz6.

In addition to the tip/stalk pattern selection at the onset of sprouting, the final vascular network is strongly influenced by the tip/stalk shuffling rate [22,23,90]. Our recent *in vitro* experiments have shown that LFNG KD cells are more prone to shuffle positions within the sprout [29]. Given the interaction of ALK1 and YAP/TAZ for the regulation of LFNG, following a similar approach as in Venkatraman et al. [62], we here investigated the ability of ALK1 KO cells to switch phenotypically (i.e., going from tip to stalk or vice versa) upon a positional change, and checked whether YAP/TAZ KO could partially counteract the ALK1-KO effects. To mimic a VEGF gradient, we initially exposed two ECs to 100% and 90% of the reference VEGF cue, ensuring the establishment of a distinct cell fate for both cells. After 24 hours, to mimic a positional switch, the external stimuli (DLL4 and VEGF) were inverted (Fig 4A). Given the possible variations in and the effects of the steepness of the VEGF gradient for the resulting potential phenotypic switch, we varied the lower level of VEGF sensed (between 10–90% of the amount of VEGF the other cell senses, see methods, equation 11).

The model predicted that, following a positional switch, wild-type ECs were able to relatively quickly switch phenotype, independent of the difference in the amount of sensed VEGF (Fig 4B and 4C). In contrast, ALK1 KO cells switched phenotype at a generally lower rate compared to wild type, and only if exposed to VEGF values differing at least a factor 2, representing relatively steep VEGF gradients. This is consistent with the impaired ability of ALK1 KO cells to repress the tip phenotype in their neighbors due to decreased LFNG expression. This behavior was partially rescued by YAP/TAZ KO. For all cases in which one cell sensed at least 20% of the amount of VEGF the other cell was sensing, the three conditions resulted in significantly different phenotypic switch rates (p < 0.001) (Fig 4B). Cells switched faster for cases in which one of the cells sensed 30–50% of the amount of VEGF the other cell was sensing. More importantly, when one cell sensed 60% of the amount of VEGF sensed by the other cell, the phenotypic switch that was inhibited by ALK1 KO was instead rescued by YAP/TAZ KO. Representative simulations showing the latter case are provided in Fig 4C, depicting filopodia activity over time. Even though the ALK1 KO ECs showed a clear decrease in the amount of filopodia for the initial tip cell upon the shuffling cue, the initial stalk cell was unable to repress the tip phenotype in its neighbor due to low LFNG and thus weakened lateral inhibition, causing the cells to maintain their original phenotypes (Fig 4C). YAP/TAZ KO could counteract this effect; in these simulations, the positional shuffling was followed by an initial decrease in filopodia expression in the original tip cell, prior to this cell adopting the stalk phenotype and the other cell taking on a tip phenotype (Fig 4C). This was (at least partially) caused by increased LFNG, partially restoring lateral inhibition potential. These simulations therefore indicate that ALK1 KO ECs maintain a high migratory behavior (indicated by high filopodia activity) also when they lose the tip position, consistent with increased positional switching observed in LFNG KO experiments [29]. Moreover, the simulations suggest that YAP/TAZ KO may partially rescue the physiological phenotypic switching potential.

### ALK1 KO primarily affects EC behavior in low stiffness regimes

**Fig 5:**
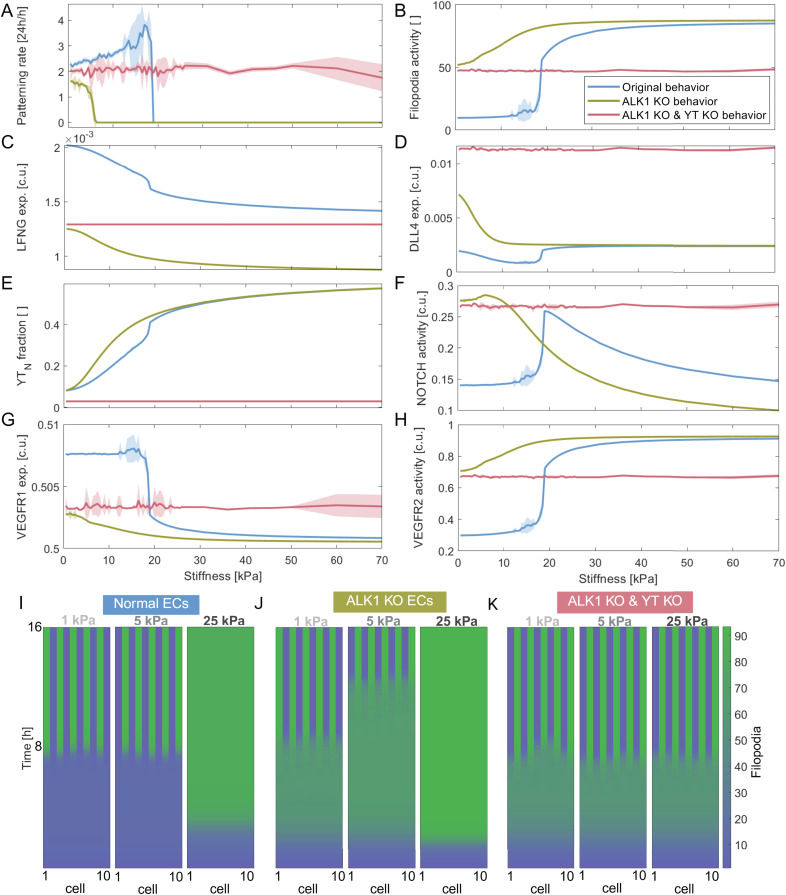
YAP/TAZ inhibition can rescue EC behavior in ALK1 KO cases, which is mainly affected in low stiffness regimes. The simulations predicted increased patterning rate **(A)**, filopodia activity **(B)**, DLL4 expression **(D)**, YAP/TAZ nuclear fraction **(E)**, NOTCH activity **(F)** and VEGFR2 activity **(H)** for ALK1 KO ECs (yellow) compared to wild-type cells (light blue), while LFNG expression **(C)** and VEGFR1 expression **(G)** decreased. Protein levels remained stable for all stiffnesses upon ALK1 KO & YT KO (pink graphs). All simulations were repeated 25 times, here showing the average with standard deviation in shaded color. For panels B-H, mean activity or expression levels were determined by averaging over the timepoints before patterning occurred (see methods). Representative time course analyses are shown for filopodia activity at three different stiffnesses; 1 kPa, 5 kPa and 25 kPa, for normal ECs **(I)**, ALK1 KO ECs **(J)** and ALK1 KO with YAP/TAZ KO ECs **(K)**. Mainly normal and ALK1 KO ECs show increasing filopodia activity with increasing stiffness. Created in BioRender. Passier, **M.** (2026) https://biorender.com/kh95tww.

YAP/TAZ nuclear translocation and NOTCH activity are known to respectively increase and decrease in response to stiffness [30,44,79,91]. Therefore, motivated by the interaction between YAP/TAZ and NOTCH as well as NOTCH with ALK1, we next examined the dependence of ALK1 KO EC behavior on different stiffnesses. For low stiffnesses, the model predicted decreased patterning rates for ALK1 KO compared to wild type. Patterning of ALK1 KO ECs ceased for stiffnesses higher than 6 kPa, a threshold much lower compared to wild-type ECs (19 kPa, Fig 5A). For stiffnesses above 19 kPa, wild type and ALK1 KO EC behavior was relatively similar, although wild-type ECs were a bit slower to all adopt the tip phenotype (Fig 5I and 5J). Additionally, the model predicted increased filopodia activity prior to pattern formation, especially for stiffnesses lower than 19 kPa (Fig 5B). The combination of these two behaviors in ALK1 KO ECs for low stiffnesses (until ceased patterning at 6 kPa) corresponds to hypersprouting, consistent with the behavior observed for ALK1 KO ECs on liver stiffness (Fig 2).

Part of this slower patterning response can be explained through LFNG, which showed significantly lower expression in ALK KO ECs throughout the whole range of simulated stiffnesses, thereby weakening lateral inhibition (Fig 5C). For both wild-type and ALK1 KO ECs, LFNG (and DLL4, Fig 5D) expression decreased for increasing stiffness, resulting from increased nuclear fractions of YAP/TAZ (Fig 5E). Similar to the behavior described for filopodia activity, DLL4 expression, the YAP/TAZ nuclear fraction, NICD and VEGFR2 activity all increased upon ALK1 KO for relatively low stiffness, most significantly below 19 kPa (stiffness for which wild-type ECs ceased patterning) (Fig 5D-5F and 5J). The concurrent elevation of these factors can be explained by the positive feedback loop leading to amplified VEGFR2 activity and consequently increased HE activity, as mentioned earlier (Fig 3B). All behaviors, except for NOTCH activity, were predicted to get closer to wild-type levels for stiffnesses above 19 kPa, while NOTCH activity went below wild-type expression levels (Fig 5F). This latter trend could result from weakened lateral inhibition due to a sharp decrease in LFNG, which we observed perhaps because of the steep increase in nuclear YAP/TAZ at approximately 19 kPa. The stiffness response of YAP/TAZ is believed to be extra sensitive in ALK1 KO ECs, due to its implementation as a Hill function (see equation S25) which led to a more rapid increase in these parameters under ALK1 KO conditions, as modulated by the VEGF-FAK interaction. Interestingly, the largest behavioral differences were all observed for the low stiffness regions, corresponding to stiffnesses associated with organs frequently affected by AVM formation in HHT2 patients [2,4,8,50,92,93].

Finally, we checked how YAP/TAZ KO affected behavior of ALK1 KO ECs for different stiffnesses. Not surprisingly, YAP/TAZ KO caused ECs to lose (most of) their stiffness-dependent behavior. As a result, this caused the expression of DLL4 and LFNG (Fig 5C-5E), and therefore VEGFR2, VEGFR1 and NOTCH activities (Fig 5F-5H), to remain at approximately the same level for all stiffnesses. This resulted in the patterning rate and filopodia activity pre-patterning to remain at stable levels over the whole range of stimulated stiffnesses (Fig 5A,5B and 5K), with levels similar to wild-type ECs exposed to low stiffness. This indicates that YAP/TAZ KO can (partially) rescue the physiological filopodia formation and tip/stalk patterning rate for ALK1 KO ECs, independently of stiffness.

### Knockdown of cytoskeletal elements decreases YAP/TAZ signaling and rescues patterning in ALK1 KO ECs for higher stiffnesses

**Fig 6:**
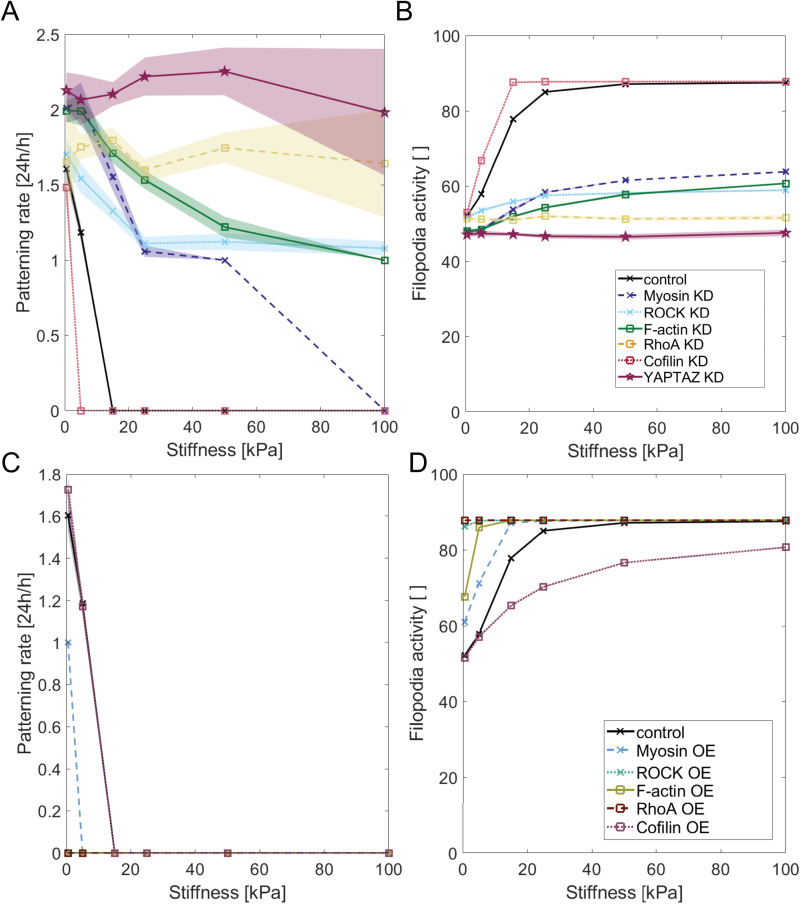
Downregulation of cytoskeletal elements can rescue patterning and reduce filopodia activity in ALK1 KO ECs. Patterning rates **(A)** and average filopodia activity prior to pattern formation **(B)** for downregulation (DR) of several cytoskeletal elements: myosin, ROCK, F-actin, RhoA, Cofilin and YAP/TAZ. Upregulation (UR) of the same cytoskeletal elements leads to inhibited patterning **(C)** and generally increased filopodia activity **(D)**. All simulations were repeated 25 times. The mean and standard deviation (shaded colors) are shown. Created in BioRender. Passier, **M.** (2026) https://biorender.com/b4ceqt6.

Simulations with our original model suggested that cytoskeletal perturbations can regulate tip/stalk dynamics by modulating YAP/TAZ activity [45]. Therefore, we next investigated whether perturbing cytoskeletal elements could rescue phenotypic selection (temporal) dynamics of ALK1 KO ECs. For all inhibitors except Cofilin, due to its inhibitory relationship with YAP/TAZ, cytoskeletal downregulation rescued patterning in ALK1 KO ECs for stiffnesses above 6 kPa (Fig 6A). Downregulation of the cytoskeletal elements even enabled patterning of ALK1 KO ECs for stiffnesses beyond which patterning normally ceased for wild-type ECs (Fig 6A and 5A). The simulations indicated that targeting elements more upstream in the signaling cascade provided the largest effect (more closely mimicking that of direct YAP/TAZ targeting). Interestingly, no patterning was observed at 100 kPa for the simulation involving the myosin inhibitor, suggesting that myosin is a less potent target in the prevention of AVM formation. For most inhibitors, filopodia activity stabilized at relatively high levels, similar to that of ALK1 KO ECs for low stiffnesses (Fig 6B).

In the case of upregulated cytoskeletal elements (Fig 6C-6D), none of the simulated cases patterned beyond the original stiffness threshold; patterning was actually inhibited in most cases (Fig 6C). This trend was accompanied by high filopodia activity throughout the whole range of simulated stiffnesses (Fig 6D). This behavior for upregulated cytoskeletal elements led to high nuclear YAP/TAZ for low stiffness levels onwards, low LFNG and DLL4 (and overall NICD) activity, in combination with high VEGFR2 expression, leading to decreased patterning and increased filopodia activity.

## Discussion

The processes underlying HHT2, involving a complex interplay of signaling pathways that collectively contribute to aberrant angiogenesis, remain incompletely characterized. This may have detrimental consequences for patients. In the present study, we specifically investigated the roles of YAP/TAZ stiffness mechanosensitivity and YAP/TAZ interplay with the NOTCH pathway in the context of angiogenesis dysregulated by ALK1 KO, known to cause HHT2. Upon ALK1 KO, our simulations showed increased nuclear YAP/TAZ and slow phenotypic pattern formation accompanied by high filopodia activity prior to pattern formation, indicating hypersprouting. As a consequence of YAP/TAZ stiffness sensitivity, these behaviors were mainly observed for low stiffnesses, whereas ALK1 KO cells more closely resembled wild-type behavior on higher stiffnesses. Moreover, the simulations indicated that ALK1 KO ECs were less likely to switch phenotype upon a positional shuffle compared to wild type, suggesting more migratory behavior. Furthermore, the simulations predicted that either direct or indirect (via cytoskeletal elements) inhibition of YAP/TAZ could partially restore EC signaling and angiogenesis temporal dynamics, thus potentially attenuating the formation of aberrant vasculature due to disturbed phenotypic selection.

Previous computational studies have also used computational modelling to investigate EC behavior in HHT2, focusing on the EC polarization response to the mechanical regulator flow, and suggested junctional dysfunction as a new treatment target to prevent AVM formation [94]. The critical role of temporal dynamics of signaling pathways has been gaining increasing attention, also in the field of pathogenesis, e.g., highlighting the critical role of LFNG, using *in silico* methods [29,46,47]. In this study, we took those temporal dynamics into account and focused mainly on phenotypic selection, and the effect of stiffness, another important mechanical cue impacting YAP/TAZ signaling.

Our results are consistent with a recent experimental study showing that YAP/TAZ signaling may play a crucial role in HHT2 [6]. In their experiments, ALK1 KO led to pronounced upregulation of nuclear YAP/TAZ and to impaired endothelial polarization against the flow [6]. This was restored by YAP/TAZ inhibition, which also prevented AVM formation [6]. Since it has been shown that AVM formation is initiated by aberrant sprouting from veins [15], we hypothesized that targeting YAP/TAZ signaling due to its interactions with NOTCH, can also rescue the aberrant tip/stalk temporal dynamics caused by ALK1 KO. Our simulations predicted that ALK1 KO caused increased nuclear YAP/TAZ and VEGFR2 activity, and decreased LFNG expression and NOTCH activation, in agreement with experiments [6,25,26]. The predicted hypersprouting is corroborated by observations in multiple experimental models [5,6,29]. Therefore, the identified signaling interactions accurately describe and explain several experimental results focused on HHT.

In our simulations, inhibition of YAP/TAZ in ALK1 KO ECs led to faster patterning, in combination with lower filopodia, VEGFR2, and NOTCH activity. So, it partially normalized phenotypic selection of ALK1 KO ECs and potentially reduced hypersprouting, in spite of the protein levels not being completely restored to physiological signaling levels. This is consistent with our assumption that YAP/TAZ mainly affect LFNG and DLL4 [43–45], while the ALK1 KO mutation mainly affects LFNG and HE. Targeting cytoskeletal elements, or other regulators of YAP/TAZ nuclear translocation [95], may help to prevent hypersprouting, but could also lead to off-target effects, for example affecting ROCK, which regulates vessel permeability [96]. There should therefore be a careful trade-off between effectiveness of the chosen target and the potential off-target effects. Overall, our simulations indicate that the effects of YAP/TAZ, upregulated by the overactive VEGF-VEGFR2 signaling loop in ALK1 KO, play a major role in dysregulated phenotypic selection, that could be (partially) restored by inhibiting it. Inhibitors that target the YAP-TEAD interaction might be particularly relevant [97], especially since an agent acting through interference with the YAP-TEAD signaling axis, verteporfin [98], has been shown to prevent AVM formation in Alk1-deficient mice [6]. Given the direct interaction between YAP/TAZ - LFNG and YAP/TAZ - DLL4 in our system, LFNG and DLL4 emerge as additional targets, that could potentially be upregulated to normalize EC behavior [26,29].

Our simulations also highlight the importance of selecting multiple measurement timepoints in the experiments, given the oscillating nature of the signaling pathways involved. For example, VEGFR1 expression decreased only minimally upon ALK1 KO in our simulations, in contrast to experimental observations of Ola and Thalgott [5,25]. A possible explanation could be a difference in the measured timepoint. In support of this, their data shows a dynamic temporal profile of VEGFR2 activity, with an initial steep increase that then gradually decreases. Our simulations indicated another important temporal component, showing initially decreased expression of HE, followed by increased expression, due to the positive VEGF feedback loop. This reinforces the notion that the dynamics of angiogenesis are characterized by temporal fluctuations that need to be taken into account in study design [46,55,61].

Environmental stiffness is another important mechanoregulator of angiogenic sprouting activity, in addition to flow mechanosensitivity [91]. Our simulations indicated a shift in stiffness-responsive behavior of ALK1 KO ECs compared to wild-type ECs, with major effects in low stiffness regions specifically. This may explain why AVMs often occur in softer tissues, such as the liver, brain and lungs [2,4,8], all with stiffnesses generally below 6 kPa [50,51,85,99–101]. Wild-type angiogenic sprouting has been shown to be optimal for intermediate stiffnesses, characterized by fast phenotypic patterning and dense vascular networks [46,47,83,84,101]. Nevertheless, these previous experimental studies have identified different values of optimal intermediate stiffness, most likely due to different experimental conditions [83,84]. Together with the known differences across ECs from different sources [102–104], and therefore organs, this indicates that while the trends identified by our simulations (performed taking experiments on HUVECs as a reference) are most likely conserved, the specific thresholds and value of optimal stiffness may vary across EC sources and organs. The main parameters to fit against organotypic endothelial data in a mechanobiological study would include those describing FAK phosphorylation. First evidence suggests inhibition of FAK can partially negate the distinct responses of arterial and venous ECs to different ECM conditions (e.g., coatings) [105], implicating FAK as a key regulator. Additionally, FAK activity is a sensitive model component, strongly dictating the YAP/TAZ nuclear translocation response. Other potential parameters that may vary include those governing RhoA activity. This possibility is supported by the regulatory function of RhoA in vascular permeability [106], a property that varies substantially between endothelium subtypes [107], and across different organs [108].

Interestingly, experiments have shown that sprouting ECs contribute to a collection of rather heterogeneous stiffnesses in the sprout environment, potentially enhancing their own sprouting ability, as corroborated by our own previous simulations [45,109]. This process might be dysregulated in HHT2 too; in fact, some ECs involved in vascular malformations secrete higher amounts of MMPs, leading to changes in stiffness and increased degradation of the extracellular environment thereby potentiating excessive sprouting [110,111]. In addition to this, fluid shear stress has recently been suggested to play a major role in HHT2 through the mechanosensitive ion channel PIEZO1 [112–115]. Degradability, tissue stiffness and flow shear stress, are all mechanical cues shown to affect angiogenesis in HHT, further supporting the notion of mechanical stimuli as important contributors to the HHT2 pathology.

Cells with impaired LFNG signaling, such as ALK1 KO cells, showed increased positional shuffling in experiments [29], corroborated by the increased filopodia activity observed in our simulations. However, despite positional switching, our simulations indicated decreased phenotypical shuffling for ALK1 KO. Earlier studies have described and investigated this phenotypical shuffling and suggested the VEGFR1/VEGFR2 balance to be critical for a normal phenotypic switch [22]. Our simulations predicted a disturbed balance with highly elevated VEGFR2 activation and minor changes to VEGFR1 activity, which could be one of the causes for the observed impaired shuffling, potentially through impairment of lateral inhibition. Phenotypic shuffling is required for physiological network formation, and previous experiments indicated that if phenotypic switching is reduced, this could lead to less branching (and potentially expansion instead of branching) [47]. Hence, the decreased phenotypic switching capacity observed in the simulations indicates decreased phenotypic shuffling rates in ALK1 KO ECs, leading to less branching and instead vessel expansion, in agreement with vessel dilatation observed in HHT2 [2,116]. This observation of decreased phenotypic switching potential in ALK1 KO ECs is also supported by experiments indicating ectopic expression of arterial markers in veins that show signs of premature AVMs in mice [117]. *In vitro*, VEGF needs to become inactive for ECs to achieve a venous phenotype [118]. This might not be possible in ALK1 KO ECs, due to the overactive VEGF-VEGFR2-filopodia positive feedback loop. ALK1 KO ECs may have impaired differentiation capacity, preventing them from correctly forming all the endothelial subtypes necessary to obtain a healthy capillary bed. In later stages too, after tip/stalk pattern establishment, it was observed that LFNG-KO ECs did not return to their quiescent state [29]. In summary, these findings suggest that ALK1 KO tip cells remain hyperactive, potentially unable to regulate NOTCH and VEGF activity, thereby unable to differentiate between the different phenotypes, leading to aberrant sprouting and erroneous arterio-venous connections. This could potentially be alleviated by YAP/TAZ inhibition, as the simulations indicated (partially) increased phenotypic switching potential in some settings.

In the present study, we restricted our simulations to tip-stalk patterning before sprout formation and to tip-stalk phenotypic shuffling when the sprouts are already formed. ALK1 and YAP/TAZ most likely heavily impact other angiogenesis steps, such as sprout formation and anastomosis [43,119–121]. During these steps, ECs undergo dramatic changes in cell shape and cell-cell contacts, which are known to significantly influence YAP/TAZ [122–125] and NOTCH dynamics [126–128]. Explicitly modeling the mechanosensitive nuclear-pore opening and the influence of nuclear deformation on YAP/TAZ translocation instead of using stress fibers as a proxy could provide more handles to tune YAP/TAZ activity, supported by earlier findings that these processes contribute to the YAP/TAZ stiffness-response [129,130]. To account for such nuclear or cellular changes in shape, future computational studies could build on our work by coupling the system of ODEs to an agent-based modelling framework [66,131–133], allowing for exploration of spatial features of cells and their environment. Specifically, the effects of stiffness on YAP/TAZ in unison with the effects of temporal changes in cell shapes and cell-cell contacts on tip-stalk phenotypic selection during sprout formation could then be investigated.

In summary, the developed modelling framework has allowed us to investigate the phenotypic selection and shuffling behavior of ALK1 KO ECs over a range of stiffnesses, with and without YAP/TAZ inhibition. Our results highlight that ALK1 KO impairs phenotypic selection and shuffling by inhibiting NOTCH1 activation, especially in low stiffness environments, and indicate that targeting YAP/TAZ and endothelial stiffness sensitivity might provide promising therapeutic strategies to restore the physiological dynamics of angiogenesis.

## Methods

To elucidate the mechanisms underlying aberrant phenotypic selection by ECs in HHT2, we expanded our previously established modelling framework describing VEGF-NOTCH1 signaling and its crosstalk with YAP/TAZ in a 1D series of communicating ECs [29,45]. More specifically, we extended the set of ordinary differential equations (ODEs) with the VEGF-FAK interaction, to allow for bidirectional feedback and split the generic VEGFR in the model into VEGFR1 and VEGFR2.

### Main model assumptions

**Fig 7:**
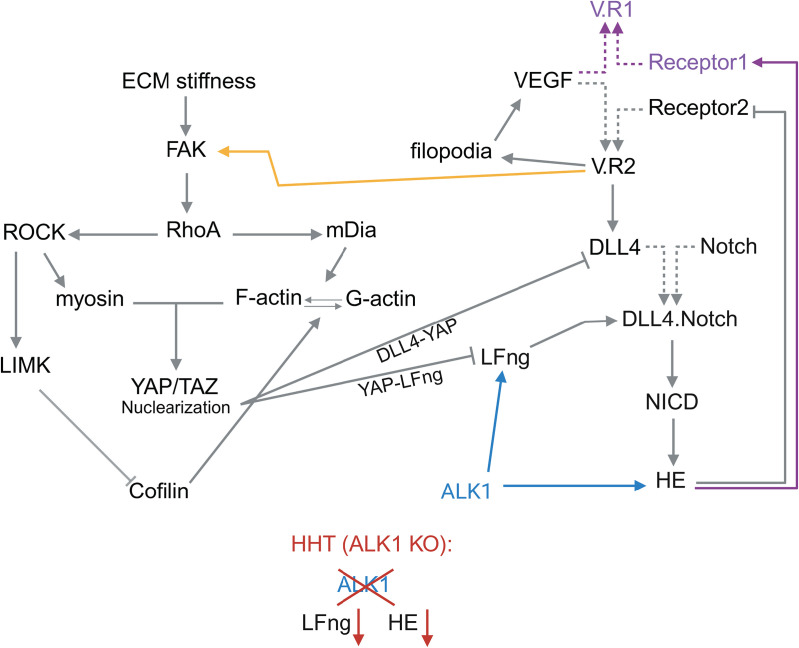
Schematic overview of the modeling framework. In gray all interactions present in the previous framework. In purple the interactions reflecting the split of VEGFR in VEGFR1 and VEGFR2, in yellow the interaction between VEGF and FAK, in blue the effects of ALK1 and in red those of ALK1 KO. Created in BioRender. Passier, **M.** (2026) https://biorender.com/baulwlq.

For each cell, a system of ODEs was considered, with each single ODE representing a component present in the model schematic (Fig 7). In the case of DLL4, NOTCH1, and their bound complex, two different ODEs were adopted to track the concentrations of these membrane-bound proteins on the left and right edge of each cell. This allowed us to model and track the activity of these membrane-bound proteins in detail, considering that their interaction and temporal variation depends on the specific cell neighbor [134].

#### Original modelling framework.

In the modelled VEGF-NOTCH signaling cascade [29,62], simulated filopodia activity increases upon VEGF binding to VEGFR2, thereby increasing the VEGF sensing ability of the respective cell. At the same time, activated VEGFR2 upregulates DLL4 expression, leading to increased transactivation of NOTCH1 receptors on adjacent cells. Subsequent cleavage and translocation of the NOTCH1 Intracellular Domain (NICD) to the nucleus induces expression of target genes (HES/HEY, labelled here with HE) in those neighboring cells, which repress VEGFR2 expression, thereby decreasing their VEGF sensing abilities – a mechanism called lateral inhibition.

In Passier et al. we incorporated the mechanosensitivity of EC phenotypic selection by integrating the VEGF-NOTCH1 model with an ODE-based model describing YAP/TAZ nuclear translocation through cytoskeletal interactions [36,45]. Briefly, substrate stiffness modulates the non-linear activity and clustering of adhesion proteins, generally represented and quantified in the model by the amount of phosphorylation of Focal Adhesion Kinase (FAK). Phosphorylated FAK (pFAK) subsequently activates RhoA. RhoA signaling promotes G-actin polymerization into F-actin via mDia, and stabilizes F-actin through ROCK mediated activation of LIMK, which inhibits the F-actin severing activity of Cofilin. Additionally, ROCK enhances myosin contractility, and together with F-actin, leads to the formation of contractile (actomyosin) stress fibers. These stress fibers are assumed to induce nuclear flattening – though not explicitly modelled - which enhances YAP/TAZ nuclear translocation [35,36]. This occurs in synergy with YAP/TAZ regulation through canonical Hippo signaling, implemented through the modelled LATS-LIMK interaction. Importantly, the YAP/TAZ and NOTCH1 pathway are coupled via YAP/TAZ-mediated inhibition of both DLL4 and LFNG expression [43,44,135]. This coupling enables mechanical cues, such as stiffness, to modulate VEGF-NOTCH1 signaling and endothelial phenotypic selection dynamics [45].

#### Extended model.

Previous studies have included the effect of BMP9 by its upregulation of HE as well as LFNG [29]. We used this to include ALK1, and to mimic the ALK1 KO mutation. We build on top of these studies by implementation of VEGF-FAK, to include the bidirectional crosstalk between the YAP/TAZ and VEGF-NOTCH pathways, which we hypothesize to be essential for both physiological as well as pathological angiogenesis, including that in HHT2 patients. Both stiffness as well as VEGF-dependent FAK activation rely on integrin interactions [136,137]. At the same time, both enhance integrin activity too [138,139]. Since integrins are not explicitly modeled in our framework, we assume a direct interaction between VEGF and FAK phosphorylation, that synergizes with the direct interaction between stiffness and FAK, thereby upregulating nuclear YAP/TAZ [41,42,72,73]. Moreover, we adapted the parameters of the VEGFR parameter in the original model to reflect solely VEGFR2 behavior, and included VEGFR1, that acts as a decoy receptor, is generally much more abundantly present than VEGFR2 [71] and is upregulated by HE [17,19,22]. Finally, we have adapted the boundary conditions and model equations to simulate EC phenotypic shuffling, occurring after the initial tip-stalk selection and sprout formation [22]. For all model interactions, see Fig 7. In this section we highlight the modified and extended equations. For a complete overview of all model equations, please refer to S1 Text.

### VEGF-mediated FAK phosphorylation

The original equation for FAK (φ) phosphorylation [36,45]was expanded to include active VEGFR2 signaling (${V.R}_{\text{2}}$) as an additional modulator that synergizes with the effects of stiffness (E):


$$\begin{array}{c} \;\frac{d\varphi }{dt}=k_{sfdf}\;\frac{{\left(E{\left(\text{1}+V.R_{\text{2}}\right)}^{n_{\text{2}}}\right)}^{n_{\text{1}}}}{{C_{\varphi }\;+\;\left(E{\left(\text{1}+V.R_{\text{2}}\right)}^{n_{\text{2}}}\right)}^{n_{\text{1}}}}\;\left(\varphi_{\text{0}}-\varphi \right)-\varphi \end{array}$$ (1)

in which $\varphi_{\text{0}}$ denotes the total amount of FAK, $k_{sfdf}$ represents the net rate of FAK activation and dephosphorylation and $C_{\varphi }$ acts as a Michaelis-Menten-like constant, indicating the inflection point where there is a shift in behavior resulting from the combined effect of the pro-adhesion formation stimuli – stiffness (E) and activated VEGFR2 ($V.R_{\text{2}}$) – for which the phosphorylation rate of FAK reaches half of its maximum. $V.R_{\text{2}}$ was introduced in a $\text{1}+{V.R}_{\text{2}}$ formulation, to ensure that stiffness can still induce YAP/TAZ nuclear translocation independently of VEGF [140]. Moreover, a distinct exponent ($n_{\text{2}}$) was applied to the ${V.R}_{\text{2}}$ term to capture the experimentally observed non-linear dependence of phosphorylated FAK on VEGF [41]. FAK phosphorylation driven by mechanical cues such as substrate stiffness relies on integrins, which provide the essential connection between the ECM and cytoskeleton [141,142]. Similarly, VEGF-induced FAK phosphorylation requires integrin signaling [75–77]. These experimental observations suggest a synergistic interaction between VEGF signaling and mechanical input in regulating phosphorylation of FAK, with both regulations requiring and converging on integrin signalling. Based on this, we have incorporated VEGFR2 as a stiffness-dependent synergistic modulator of FAK phosphorylation, consistent with the assumption that VEGFR2 effects on FAK are (much) lower when no or few integrins are present due to low stiffness [78]. The model then captures that stiffness and VEGF driven upregulation of FAK phosphorylation promote actin-myosin activity and stress fiber formation, which deform the nucleus and stretch nuclear pores, thereby potentiating YAP/TAZ nuclear translocation [35,143].

As a result of this implemented feedback mechanism, nuclear YAP/TAZ levels can become heterogeneous across the row of ECs, in contrast to their previous homogeneous distribution. Given that nuclear YAP/TAZ modulates the binding affinity between DLL4 and NOTCH1, this introduces heterogeneity with respect to binding affinities too. Moreover, to accurately reflect interactions on both cell edges, we distinguish between the left and right part of each modelled EC, allowing for spatially resolved NOTCH1-DLL4 interactions on both edges. Consequently, cell i interacts with cell i−1 on its left side and with cell i+1 on its right side. Consistent with the previously described mechanism of Fringe-mediated glycosylation of the NOTCH1 receptor determining the binding affinity [27,28], we assign the binding affinity ($k_{{\text{2}}_{i}}$) of the NOTCH1 receptor-bearing cell. Accordingly, when DLL4 on the left side of cell i interacts with NOTCH1 on the right side of cell i−1, the governing binding affinity is that of cell i−1. An example below:


$$\begin{array}{c} {\frac{dD_{L_{i}}}{dt}=\;\frac{\text{1}}{\text{2}}D}_{p_{i}}X_{{yt}_{i}}-\;k_{{\text{2}}_{i-\text{1}}}D_{L_{i}}N_{R_{i-\text{1}}}+\;k_{-\text{2}}D.N_{R_{i-\text{1}}}+W\left(\frac{D_{L_{i}}+D_{R_{i}}}{\text{2}}-\;D_{L_{i}}\right)-\phi D_{L_{i}} \end{array}$$ (2)

$$\begin{array}{c} k_{{\text{2}}_{i-\text{1}}}=\;k_{\text{2}}L_{F}(Y_{N_{i-\text{1}}}) \end{array}$$ (3)

$$\begin{array}{c} D_{p_{i}}=\;\beta_{D}+\frac{\theta V.R_{\text{2}i}^{\text{2}}}{\text{1}+V.R_{\text{2}i}^{\text{2}}};\;X_{{yt}_{i}}=\lambda +\frac{\text{1}-\;\lambda }{\text{1}+\;{\left(\frac{Y_{Ni}}{Y_{Y\text{0}}}\right)}^{n}} \end{array}$$ (4)

where $D_{p}$ represents the production of DLL4, determined by the basal production rate ($\beta_{D}$) and the stimulatory effect of activated ${V.R}_{\text{2}}$, scaled by θ. This term is multiplied with $X_{{yt}_{i}}$ to account for the repressing effect of YAP/TAZ, governed by the amount of nuclear YAP/TAZ ($Y_{N_{i}}$), a baseline value of nuclear YAP/TAZ ($Y_{Y\text{0}}$), a scaling factor (n) and λ, determining the maximum effect of this term (e.g., an λ of 0.5 indicates a maximum repressing effect of 50%). $k_{{\text{2}}_{i-\text{1}}}$ indicates the binding affinity of DLL4 on the left side of cell i to bind with NOTCH on the right side of cell i−1 ($N_{R_{i-\text{1}}}$), as determined by the amount of nuclear YAP/TAZ in that cell ($Y_{N_{i-\text{1}}}$), a basal value for the binding affinity ($k_{\text{2}}$) and LFNG, linearly taken into account by $L_{F}$. Bound DLL4-NOTCH (${D.N}_{R_{i-\text{1}}}$) can also dissociate, with probability $k_{-\text{2}}$. Additionally, W represents diffusion of DLL4 among the left and right cell edges and ϕ indicates degradation of DLL4.

### Implementation of the differential roles of VEGFR1 and VEGFR2

In addition to incorporating the VEGF-FAK interaction, we extended the model by differentiating between VEGFR2 and VEGFR1. To account for competition of VEGF-receptor binding, reflecting the spatial and availability constraints, we introduced the ratio (ξ) between VEGFR1 ($R_{\text{1}}$) and VEGFR2 ($R_{\text{2}}$), representing the generally relative abundance of $R_{\text{1}}$ compared to $R_{\text{2}}$ [69,71]. This ratio is incorporated as a scaling factor modulating the effective binding rate ($k_{\text{1}}$) of VEGF to its respective receptors, to compensate for the fact that we do not have spatial details in the model framework, but still capture VEGFR1-VEGFR2 competition for VEGF-binding in a simplified, phenomenological manner. Since we assume, as many others in the field [23,29,56,60,62,134,144], that VEGF is constant over time, mass-action kinetics would fail to reproduce VEGFR1-VEGFR2 competition, as VEGF would never be depleted. To capture that the presence of VEGFR1 limits the availability of VEGF to VEGFR2, we have adopted this phenomenological competition term (the first term of equations 6–9 on the right-hand side). Moreover, the factor $R_{\text{2}}^{\text{2}}$ reflects the known cooperativity of VEGFR2 dimerization and clustering required for signaling activation (consistent with [145–147]). This gives rise to the following formulas:


$$\begin{array}{c} \xi_{R_{\text{1}}}=\frac{R_{\text{1}}}{\left(R_{\text{1}}+R_{\text{2}}\right)};\;\xi_{R_{\text{2}}}=\frac{R_{\text{2}}}{\left(R_{\text{1}}+R_{\text{2}}\right)} \end{array}$$ (5)

$$\begin{array}{c} \frac{dR_{{\text{1}}_{i}}}{dt}={-k}_{{\text{1}}_{\text{1}}}V.R_{{\text{1}}_{i}}\xi_{R_{\text{1}}}+k_{{-\text{1}}_{\text{1}}}V.R_{{\text{1}}_{i}}-\Phi_{R_{\text{1}}}R_{{\text{1}}_{i}}+\gamma_{R_{\text{1}}}+\frac{k_{up\text{1}}R_{{\text{1}}_{i}}H_{i}^{\text{2}}}{\text{1}+H_{i}^{\text{2}}} \end{array}$$ (6)

$$\begin{array}{c} \frac{dR_{{\text{2}}_{i}}}{dt}={-k}_{{\text{1}}_{\text{2}}}V.R_{{\text{2}}_{i}}\xi_{R_{\text{2}}}+k_{{-\text{1}}_{\text{2}}}V.R_{\text{2}}-\Phi_{R_{\text{2}}}R_{{\text{2}}_{i}}+\gamma_{R_{\text{2}}}-k_{inh}R_{{\text{2}}_{i}}H_{i}^{\text{2}} \end{array}$$ (7)

$$\begin{array}{c} \frac{d{V.R}_{{\text{1}}_{i}}}{dt}=\;k_{{\text{1}}_{\text{1}}}{V.R}_{{\text{1}}_{i}}\xi_{R_{\text{1}}}-k_{{-\text{1}}_{\text{1}}}V.R_{{\text{1}}_{i}}-\;\Phi {V.R}_{{\text{1}}_{i}} \end{array}$$ (8)

$$\begin{array}{c} \frac{d{V.R}_{{\text{2}}_{i}}}{dt}=\;k_{{\text{1}}_{\text{2}}}{V.R}_{{\text{2}}_{i}}\xi_{R_{\text{2}}}-k_{{-\text{1}}_{\text{2}}}V.R_{{\text{2}}_{i}}-\;\Phi {V.R}_{{\text{2}}_{i}} \end{array}$$ (9)

in which $k_{-\text{1}}$ denotes the dissociation rate of the VEGF-Receptor complex (V.R), Φ represents the degradation rate and γ indicates the basal production rate. By distinguishing between $R_{\text{1}}$and $R_{\text{2}}$, we can explicitly implement the different regulatory effects of NOTCH target genes (H) on the different receptors. More precisely, H upregulates $R_{\text{1}}$ expression (via $k_{up\text{1}}$), which is implemented analogously to the downregulatory effect of H on $R_{\text{2}}$ (via $k_{inh}$). To prevent excessive accumulation and numerical instability of unbound $R_{\text{1}}$, the upregulatory term is divided by an additional H-dependent denominator, analogous to the formulation used for DLL4 upregulation upon V.R activation.

### Mimicking ALK1 KO

In correspondence with previous work [29], we implemented the effects of BMP9 signaling through ALK1, by implementation of a parameter affecting both LFNG, and through that, the binding affinity ($k_{{\text{2}}_{i}}$), as well as the HE basal production rate ($\beta_{HE}$). In case of ALK1 KO (HHT2), this parameter ($B_{eff}$), decreases to below one to lower LFNG and HE activity, while in case of BMP9 stimulation, this parameter is set to a value above one:


$$\begin{array}{c} k_{{\text{2}}_{i}}=\;k_{\text{2}}\;YY\left(Y_{N_{i}}\right)\;B_{eff};\beta_{HE}=\beta_{HE}\;B_{eff} \end{array}$$ (10)

We opted for an implicit implementation of BMP9/ALK1 signaling, as this allowed us to predict tip-stalk dynamics in physiological, ALK1 KO and YAP/TAZ KO conditions, which was the focus of our study. Additionally, previous work demonstrated that this implicit implementation is sufficient to reproduce *in vitro* and *in vivo* experimental observations of tip-stalk dynamics subsequent to ALK1-BMP9 signaling manipulations [29].

### Parameter choices & fitting

Most of the parameters already present in the model were retained from previous studies [29,36,45,62]. However, specific parameters related to FAK phosphorylation (see equation 1) were adjusted to accommodate the addition of the VEGF-FAK interaction, while maintaining consistency with previously captured and validated model behaviors. More specifically, the net activation/dephosphorylation rate ($k_{sfdf}$), Michaelis-Menten constant (C) and the exponent specifically for the ($\text{1}+{V.R}_{\text{2}}$) term ($n_{\text{2}}$) were fitted to experimental data obtained by Abedi et al., who observed increasing FAK phosphorylation with increasing VEGF concentrations [41] and experimental data showing increasing nuclear YAP/TAZ for increasing stiffness [79]. Initial parameter estimation was conducted using the least-squares non-linear fitting algorithm of MATLAB (lsqnonlin). Given the dependency of the fitted values on the initial estimates, the algorithm was repeated 15 independent times, every time with with different initial estimates that were randomly generated between an upper and lower boundary (S2 Table). For each parameter, the repeated rounds of optimization identified parameter values, here labelled with $v_{\text{1}},\ldots ,v_{\text{1}\text{5}}$, from which we chose the value that minimized the discrepancy between the model output and experimentally observed FAK phosphorylation for increasing VEGF levels, and YAP/TAZ nuclear translocation for increasing stiffness. The resulting model predictions (S2 Fig) could replicate the increasing trends of FAK phosphorylation in response to VEGF, and YAP nuclearization in response to stiffness. However, quantitative differences could be observed, consistent with the large variability observed in different studies for YAP nuclear/cytoplasmic ratio [6,79,123]. Given this observed variability, we focused on capturing the crucial trends, rather than specific quantifications. In experimental observations [79], it can be noticed that nuclear YAP/TAZ exhibits high sensitivity and an increase for low stiffness regimes (0.5 kPa to 1 kPa) and continues to increase, more gradually, for higher stiffness levels (70 kPa onwards) [79]. In addition to these conditions, we aimed for the model to reproduce the biphasic response with respect to increasing stiffnesses, with thus an optimum stiffness for phenotypic selection (at a realistic value), and should cease patterning around 24 kPa, consistent with the original model behavior and previous experimental results [45,83,84]. In addition to this, VEGF concentrations slightly above the levels used to instigate physiological patterning should yield a further increase in both phosphorylated FAK and nuclear YAP/TAZ, avoiding saturation at physiological VEGF levels. To address these additional criteria, final parameter tuning was performed manually, as in other studies [66], for $k_{sfdf}$, C, $n_{\text{2}}$ and $n_{\text{1}}$, using the initial fitted values as starting points. Only small changes were made with respect to $k_{sfdf}$ and $n_{\text{2}}$, within the range of the predicted fitted values (S1 Table). $n_{\text{1}}$ was slightly increased (from 0.5 to 0.6) to allow for realistic YAP/TAZ nuclear translocation dynamics for ECs, similar to the previous framework [45]. Finally, manual tuning of the Michaelis Menten constant, C, which is considerably context-dependent [36,148,149], was performed outside of the predicted parameter range to capture the initial steep increase in nuclear YAP/TAZ observed at low stiffnesses and a more gradual increase at higher stiffnesses, which was lost when using the fitted parameters (S2C Fig). S2A Fig compares the model predictions obtained with the formally fitted and manually tuned parameters, showing that both can capture the trend of increasing phosphorylated FAK with increasing VEGF concentrations, with little changes in predicted values resulting from parameter tuning (purple and blue bar). Additionally, the final parameters predicted a biphasic phenotypic selection response at realistic stiffnesses [83,84,91,150], which was also lost when using the formally fitted parameters (S2D Fig).

In addition to the adjustment of FAK-phosphorylation parameters, several new parameters were introduced to account for the split of the VEGF receptor into $R_{\text{1}}$and $R_{\text{2}}$. These parameters were mainly based on previous experimental studies, indicating higher binding affinity between $R_{\text{1}}$ and VEGF compared to $R_{\text{2}}$[151], increased degradation rates for $R_{\text{1}}$ compared to $R_{\text{2}}$, based on their respective half life times [152] and a larger effect of transcriptional regulation by H on $R_{\text{1}}$ than on $R_{\text{2}}$[22]. The degradation and dissociation rates of the bound $V.R_{\text{1}}$ complex were assumed to be equivalent to those of ${V.R}_{\text{2}}$. To estimate the relative production rate of $R_{\text{1}}$, we used available data reporting average ratios between $R_{\text{1}}$ and $R_{\text{2}}$ allowing us to calibrate baseline production of $R_{\text{1}}$ accordingly [69,71]. Lastly, motivated by previous experiments and similar to previous computational methods [29,60], we calibrated the baseline level of VEGF ($V_{\text{0}}$), to ensure that patterning under physiological conditions occurred in approximately 8 hours (Fig 2A).

To determine the scaling factors required to mimic the effects of ALK1 KO on the production of H and LFNG activity, we used qPCR data of Ristori et al [29]. Their study reported a significant decrease of NOTCH target genes HES/HEY (H) expression in ALK1 KO cells. Please refer to S1 Table for a complete overview of all parameter values.

### Model validation

In addition to fitting the model parameters on a wide range of available experimental data to ensure biologically accurate behavior, we performed model validation simulations against independent experimental observations, for several different scenario’s. Following each model alteration, we verified that essential behaviors captured in the original model were preserved [45]. One of the main aspects we considered was the nuclear fraction of YAP/TAZ in response to stiffness. Previous experimental studies have demonstrated that nuclear YAP/TAZ levels rise with increasing stiffness, showing an initial, sharp increase for the low stiffness regime and a more gradual increase for higher ranges of stiffness (70 kPa and up) [79]. Moreover, an essential element of the angiogenic mechanoresponse that we captured with our previous *in silico* model, is the existence of an optimal stiffness, yielding fast and robust patterning [45,83,84]. Throughout all steps, we verified that the model retained these essential mechanobiological features.

For the pathological scenario simulated in this study, HHT2 (simulated via ALK1 KO), we employed distinct experimental data to validate, as the HHT2 scenario deviates from the original, physiological model context. To this end, model output was compared with data of Park et al, who reported elevated YAP/TAZ nuclear fractions upon ALK1 KO and demonstrated that pharmacological inhibition of YAP/TAZ prevented AVM formation in ALK1 KO mice [6]. Additionally, VEGF receptor dynamics were validated against data of Ola et al, who showed that loss of ALK1 results in increased activity of VEGFR2, in spite of unchanged overall VEGFR2 levels, in combination with decreased levels of VEGFR1 [25].

### Model simulations

Following validation of the extended modelling framework, we performed a series of simulations to explore EC behavior in different settings. Firstly, we simulated ALK1 KO and analyzed how behavior of the different angiogenic signaling elements was affected. Subsequently, we simulated pharmacological inhibition of YAP/TAZ, in line with experiments conducted by Park et al [6], to assess to what extent the effect of ALK1 KO on those key signaling elements could be mitigated by YAP/TAZ inhibition. To further investigate endothelial plasticity, we examined the tendency of cells to undergo a phenotypic switch, in response to switching the environmental cue, comparing behavior between wild-type and ALK1 KO ECs. In addition to YAP/TAZ KO, we explored alternative intervention targets for their potential of restoring patterning and shuffling behavior upon down or upregulation. Moreover, we investigated the role of extracellular matrix stiffness under ALK1-deficient conditions, both in the absence and presence of interventions aimed at rescuing patterning.

All simulations were conducted using MATLAB 2024a, employing the ODEsolver ode15s. Initial conditions were set to zero for all intracellular proteins, and unless specified otherwise, simulations started with a 24h acclimatization period (the absence of external cues), after which specific stimuli, such as VEGF, were introduced, mimicking experimental protocols. To account for MATLAB’s finite precision [153], which otherwise causes reproducible patterns unrelated to the biology of the system (S3 Fig), a small random factor (${\text{1}\text{0}}^{-\text{1}\text{6}}$) was added to the VEGF input, as a source of noise to break the symmetry randomly, reflecting inherent biological VEGF sensing discrepancies between cells (e.g., due to differences in cell shape or position). The random factor was chosen to overcome MATLAB’s finite precision, while simultaneously avoiding relatively large effects on the patterning time (S3 Fig).

The patterning rate was determined by dividing the time allowed to form a pattern (24 hours) by the time required to establish a phenotypic patterning: $\frac{\text{2}\text{4}}{t_{pat}}$ analogously with previous studies [29,45].

Baseline stiffness was set to 5 kPa, to reflect physiological liver stiffness [50,51,85]. Lastly, periodic boundary conditions were imposed, to enable interaction between the first and last cell of the row, so maintaining continuity in the simulated environment.

#### Cell shuffling.

The process of phenotypic shuffling was simulated by switching to a two-cell model. Periodic boundary conditions were removed, limiting cell-cell interactions to a single interface (the right edge of cell 1 and the left edge of cell 2). As a consequence, all produced DLL4 and NOTCH1 was restricted to this one edge and intracellular diffusion over the edges was removed. The simulations were divided into two sequential 24 hour-phases: (i) exposure to VEGF, with a slight bias towards one cell (0.9*VEGF and 1.0*VEGF), to bias tip cell selection; (ii) a gradual transition (over 2 hours [22]) towards a new asymmetric VEGF distribution (see equation 11), favoring the tip phenotype in the opposite cell. The magnitude of the asymmetric VEGF cue ($\alpha_{s}$) used in phase (ii) of these simulations was systematically varied, ranging from 10 to 90% ($\alpha_{s}$ being between 0.1 and 0.9 respectively) for cell 2 ($V_{\text{0}}^{*}$) and 100% of the original VEGF cues ($V_{\text{0}}$) for cell 1 respectively, to examine its effects on phenotypic shuffling in both physiological and ALK1 KO conditions (see equation 11). In addition to this asymmetric VEGF cue, the cells were exposed to an asymmetric DLL4 cue in phase ii, representing DLL4 levels of a neighboring tip and stalk cell respectively (as if the cells had switched position and acquired new neighbors).


$$\begin{array}{c} V_{\text{0}}^{*}=V_{\text{0}}\;\alpha_{s} \end{array}$$ (11)

The phenotypic switch rate was determined by dividing the time needed for phenotypic switching (24h) by the time it took to adopt a new phenotype, analogously to the patterning rate.

#### Intervention targets.

To mimic downregulation of possible intervention targets, we multiplied the (de)activation rates of the respective elements with a factor 10, consistent with previous work [36,45]. More specifically, the deactivation rates of Myosin ($k_{dmy}$), ROCK ($k_{dROCK}$), RhoA ($k_{dp}$) and Cofilin ($k_{cr}$) were multiplied by 10, mimicking Blebbistatin, Y-27362, Rhosin and SZ-3 [154–157], while the F-actin polymerization rate ($k_{ra}$) was decreased with a factor 10 to simulate latrunculin [158]. We mimicked verteporfin, which inhibits YAP/TAZ by preventing its translocation to the nucleus and blocking YAP-TEAD formation, by setting the YAP/TAZ active nuclear translocation rate to zero, only allowing for basal nuclear translocation activity [6,159–161]. It should be noted, that in the model we do not explicitly distinguish between different ways of inhibiting or knocking out YAP/TAZ, which is why YAP/TAZ inhibition is referred to as ‘YAP/TAZ KO’. For completeness, we additionally simulated cytoskeletal upregulation by dividing the respective deactivation rates by 10, or by multiplying with 10 in case of the polymerization rate for F-actin. For all simulations, cells were exposed to the standard, symmetric, VEGF cue to instigate patterning.

#### External cues.

The effect of extracellular matrix stiffness was investigated by performing the simulations mentioned above across a broad stiffness range, from 0.5 to 250 kPa. The step size was incrementally increased for higher stiffnesses, to reflect decreasing sensitivity of YAP/TAZ nuclear translocation to stiffness. Beyond 250 kPa, no substantial changes in model outputs were observed*.*

Across all simulations, phenotypic selection time was defined and quantified consistently. ECs were classified as tip cells when their filopodia activity surpassed the threshold set at 20, while stalk cells needed to have a filopodia activity lower than this threshold, in accordance with previous studies [29,60]. A pattern was considered established when at least 40% of the ECs adopted the tip cell phenotype, without any of those tip cells being adjacent. To account for variability, all simulations were repeated 25 times. Moreover, average activity and expression of all outputs of interest were determined by averaging their amounts over the timepoints before patterning occurred.

#### Statistical analysis.

To test whether simulated outcomes differed significantly, we first conducted an ANOVA test in case the data were distributed normally, followed by Tukey’s Honest Significant Difference test to determine which specific groups differed significantly. In case of non-normally distributed data, we instead conducted a Kruskal-Wallis test, followed by Dunn’s test, to determine which groups differed significantly.

## Supporting information

S1 Fig Expression of HE (A), bound VEGFR2 (B) and filopodia (C) over time, for 15 simulations conducted for wild type cells (blue), ALK1 KO cells (yellow) and ALK1 KO cells with additional YAP/TAZ KO (red). We show the results upon VEGF exposure (started at t = 0, after 24 hours of acclimatization).
(TIFF)

S2 FigResults of manually tuned parameters (blue) compared to parameters formally fitted by the optimization algorithm (purple) with $C_{\varphi }$= 37, $k_{sfdf}$ = 0.12 and $n_{2}$ = 0.54, and the semi-quantitative data obtained by Abedi et al. and Kretschmer et al. (gray).Comparisons between the amount of normalized, phosphorylated FAK for different VEGF conditions (A), comparison between the amount of normalized nuclear YAP/TAZ for different stiffnesses (B), the amount of nuclear YAP/TAZ for different stiffnesses (C) and the patterning rate for the same range of stiffnesses (D).(TIFF)

S3 Fig The effect of small perturbations on patterning time for varying stiffnesses. Mean patterning time (indicated by the lines) with standard deviation (indicated by the shaded areas) for a random added factor to the VEGF source of: 0 (blue), 10^-8^ (yellow), 10^-17^ (red) and 10^-16^ (grey). All simulations were repeated 100 times.
(TIFF)

S1 Text A more elaborate explanation of the full ODE-based model, including the model equations. (DOCX)

S1 Table All parameter values used to obtain the data shown in the paper. (DOCX)

S2 Table The upper and lower boundaries used for the parameter fitting. (DOCX)
