# Supplementary figures and images for: Targeting stiffness-dependent YAP/TAZ restores angiogenesis dynamics impaired by ALK1 knockout in silico
Source: PLoS Comput Biol. 2026 Jul 16;22(7):e1013561. doi: 10.1371/journal.pcbi.1013561 (PMC13387619; doi:10.1371/journal.pcbi.1013561)

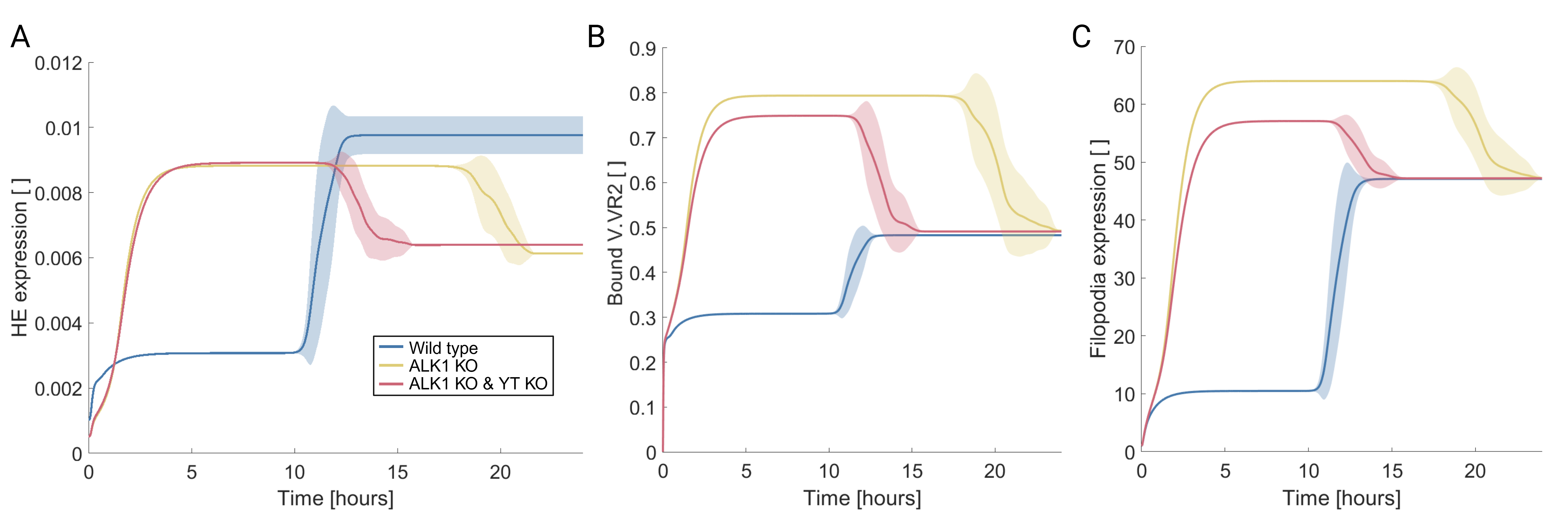

Supplement: S1 Fig — We show the results upon VEGF exposure (started at t = 0, after 24 hours of acclimatization). (TIFF) [file pcbi.1013561.s001.tiff]

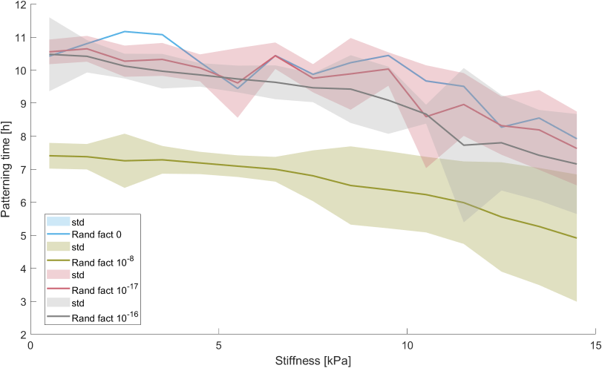

Supplement: S3 Fig — Mean patterning time (indicated by the lines) with standard deviation (indicated by the shaded areas) for a random added factor to the VEGF source of: 0 (blue), 10-8 (yellow), 10-17 (red) and 10-16 (grey). All simulations were repeated 100 times. (TIFF) [file pcbi.1013561.s003.tiff]
