# Supplementary material for: Targeting stiffness-dependent YAP/TAZ restores angiogenesis dynamics impaired by ALK1 knockout in silico
Source: PLoS Comput Biol. 2026 Jul 16;22(7):e1013561. doi: 10.1371/journal.pcbi.1013561 (PMC13387619; doi:10.1371/journal.pcbi.1013561)
Supplement: S1 Table — (DOCX) [file pcbi.1013561.s005.docx]

**S1 Table**

This section contains all parameter values used to obtain the data as shown in the paper.

| **Param.** | **Value** | **Description** | **Reference** |
| --- | --- | --- | --- |
| $V_{0}$ | 0.23 / 0.023 [cu] | Reference amount of VEGF, depending on the scenario | *Tuned to* (1,2) |
| $k_{3}$ | 0.005 [cu^-2^] | Factor scaling positive feedback between filopodia and VEGF | (2) |
| $k_{1_{2}}$ | 0.1 [cu^-1^ sec^-1^] | Rate of association of V.R2 | (3) |
| $k_{-1_{2}}$ | 0.001 [sec^-1^] | Rate of dissociation of V.R2 | (3) |
| $\phi$ | 0.005 [sec^-1^] | Protein degradation | (2) |
| $\gamma$ | 0.005 [cu sec^-1^] | Protein production | (3) |
| $k_{inh}$ | 0.003 [cu^-2^ sec^-1^] | Scales the impact of inhibition of VR2 by H | (2) |
| $\beta$ | 0.001 [cu sec^-1^] | Basal filopodia and Hes1/Hey1 formation | (3) |
| $k_{f}$ | 0.1 [sec^-1^] | Filopodia formation rate | (3) |
| $k_{-f}$ | 0.001 [sec^-1^] | Rate of filopodia turnover | (3) |
| $\theta$ | 0.1[sec^-1^] | Downregulation rate of DLL4 production by V.R | (3) |
| $k_{-2}$ | 0.1 [sec^-1^] | Dissociation rate of D.N | (3) |
| $W$ | 0.001 [sec^-1^] | Diffusion of unbound Notch and DLL4 across both cell edges | (2) |
| $k_{cat}$ | 0.1 [sec^-1^] | Catalysis rate of D.N | (3) |
| $k_{2}$ | 0.002 [cu^-1^ sec^-1^] | Association rate of D and N | (2) |
| $k_{sfdf}*$ | 0.194 [] | Net activation/dephosphorylation rate of K | *Fitted* & tuned to (4,5) |
| $\upsilon$ | 500 [] | Activation rate of RhoA, by FAK | (6,7) |
| $k_{fkp}$ | 0.018 [sec^-1^] | Activation rate of RhoA through other mechanisms than FAK | (6,7) |
| $k_{dp}$ | 0.625 [sec^-1^] | Deactivation rate | (6,8) |
| $k_{rp}$ | 2.2 [sec^-1^] | Activation rate of ROCK by RhoA | (6,9,10) |
| $k_{mp}$ | 1 [sec^-1^] | Activation rate of mDia by RhoA | (6) |
| $k_{drock}$ | 0.8 [sec^-1^] | Deactivation rate of ROCK | (6,11,12) |
| $k_{dmdia}$ | 1 [sec^-1^] | Deactivation rate of mDia | (6) |
| $k_{mr}$ | 0.015 [sec^-1^] | Activation of Myo by other pathways than ROCK | (6) |
| $k_{lr}$ | 0.07 [sec^-1^] | Activation of LIMK by other pathways than ROCK | (6,13,14) |
| $k_{dmy}$ | 0.067 [sec^-1^] | Deactivation of Myo | (6,15) |
| $k_{dl}$ | 2 [sec^-1^] | Deactivation of LIMK | (6,13,14) |
| $\varepsilon$ | 40 [] | Activation of Myo through ROCK | (6) |
| $\tau$ | 200 [] | Activation of LIMK through ROCK | (6) |
| $S_{\omega}$ | 13 [] | Smoothing parameter, determining sharpness of the activation – ROCK | (6) |
| $\omega_{s}$ | 0.26 [] | Activation threshold for the smoothing function - ROCK | (6) |
| $S_{\sigma}$ | 10 [] | Smoothing parameter, determining sharpness of the activation – mDia | (6) |
| $\sigma_{S}$ | 0.13 [] | Activation threshold for the smoothing function - mDia | (6) |
| $k_{to}$ | 0.04 [sec^-1^] | Dephosphorylation rate of cofilin | (6,13) |
| $k_{cr}$ | 0.7 [sec^-1^] | Rate of phosphorylation of cofilin by LIMK | (6,13) |
| $k_{ll}$ | 0.8 [sec^-1^] | Phosphorylation inhibition rate of cofilin by LIMK, resulting from LATS_0_ | (6,16) |
| $k_{ra}$ | 0.4 [sec^-1^] | Polymerization rate of cytoplasmic F-actin | (6) |
| $k_{dep}$ | 0.35 [sec^-1^] | Depolymerization rate of cytoplasmic F-actin | (6,17) |
| $k_{fc1}$ | 8 [sec^-1^] | Disassembly rate of F-actin by cofilin | (6,13,14) |
| $\alpha$ | 40 [] | Amplification of polymerization of F-actin due to mDia | (6,18) |
| $k_{cn}$ | 0.1 [sec^-1^] | YAP/TAZ nuclear translocation rate independent of F-actin | (6) |
| $k_{cy}$ | 20 [sec^-1^] | YAP/TAZ nuclear translocation rate dependent on F-actin | (6) |
| $k_{nc}$ | 3 [sec^-1^] | YAP/TAZ cytoplasmic translocation rate independent of LATS_0_ | (6) |
| $k_{ly}$ | 6 [sec^-1^] | YAP/TAZ cytoplasmic translocation rate dependent on LATS_0_ | (6) |
| $l_{0}$ | 0.5 | Total amount of LATS, LATS_0_ | (6) |
| $l_{p}$ | 0.05 | Phosphorylated LATS | (6) |
| $\varphi_{0}$*,* $\rho_{0}$*,* $\omega_{0}$*,* $\sigma_{0}$*,* $M_{0}$*,* $L_{0}$*,* $C_{0}$*,* $F_{0}$*,* $Y_{0}$ | 1 | Total amounts of available protein | (6) |
| $\lambda$ | 0.1 [] | Maximum inhibitory effect of $Y_{N}$ on DLL4 production | (19,20) |
| $Y_{Y0}$ | 0.47 [cu] | Fraction of $Y_{N}$ from which onwards $Y$ starts inhibiting DLL4 | (19,20) |
| $n$ | 6.5 [] | Scaling the effect of YAP/TAZ inhibition of DLL4 | (19,20) |
| $a$ | -0.6121 [] | Scaling the effect of YAP/TAZ inhibition of LFng | (4,19) |
| $b$ | 1.0578 [] | Scaling the effect of YAP/TAZ inhibition of LFng | (4,19) |
| $n_{1}$** | 0.6 [] | Adapted Hill-function exponent to capture the FAK response in endothelial cells | *Calibrated* to (4) |
| $n_{2}*$ | 1.7 [] | Exponent to capture the nonlinear interaction between VEGFR2 and FAK | *Fitted* & tuned to (4,5) |
| $C_{\varphi}$** | 13 [kPa] | $V.R2*E$ value for which activation rate equals half the maximum activation rate | *Fitted* to (4,5) |
| $k_{1_{1}}$ | 0.3 [cu^-1^ sec^-1^] | Rate of V.R1 association | *Estimated from* (21) |
| $k_{{-1}_{1}}$ | 0.001 [sec^-1^] | Rate of dissociation of V.R1 | *Assumed to be the same as VR2* |
| $ɸ_{R_{1}}$ | 0.01 [sec^-1^] | Protein degradation of VR1 | *Estimated from* (22) |
| $\gamma_{R_{1}}$ | 0.5 [cu sec^-1^] | Protein production of VR1 | *Estimated from* (23,24) |
| $k_{up1}$ | 0.0044 [cu^-2^ sec^-1^] | Scales the impact of upregulation of VR1 by H | *Estimated from* (25) |
| $B_{eff1}$ | 0.62 [] | Scaling factor of effect of ALK1 KO on LFng | (2) |
| $B_{eff2}$ | 0.5 [] | Scaling factor of effect of ALK1 KO on Hes/Hey production | (2) |

“cu” stands for concentration units.

*These values were manually tuned within the predicted parameter ranges by the fitting algorithm, to ensure a realistic EC YAP/TAZ nuclearization and biphasic patterning response (see Methods). The fitting algorithm initially predicted $k_{sfdf}$= 0.11 – 1.8 and $n_{2}$ =0.5 – 3.5.

** These values were manually tuned, outside of the predicted parameter ranges by the fitting algorithm (for $C_{\varphi}$), to ensure a realistic EC YAP/TAZ nuclearization and biphasic patterning response (see Methods). The fitting algorithm predicted $C_{\varphi}$between 29 and 44 and the previous $n_{1}$ value was 0.5.

**References**

1. Zakirov B, Charalambous G, Thuret R, Aspalter IM, Van-Vuuren K, Mead T, et al. Active perception during angiogenesis: filopodia speed up Notch selection of tip cells in silico and in vivo. Philosophical Transactions of the Royal Society B: Biological Sciences. 2021 Feb 8;376(1821):20190753. doi:10.1098/rstb.2019.0753

2. Ristori T, Thuret R, Hooker E, Quicke P, Sanlidag S, Lanthier K, et al. Bmp9 regulates Notch signaling and the temporal dynamics of angiogenesis via Lunatic Fringe. Developmental Cell. 2026 Feb 5;0(0). doi:10.1016/j.devcel.2026.01.006 PubMed PMID: 41650956.

3. Venkatraman L, Regan ER, Bentley K. Time to Decide? Dynamical Analysis Predicts Partial Tip/Stalk Patterning States Arise during Angiogenesis. PLOS ONE. 2016 Nov 15;11(11):e0166489. doi:10.1371/journal.pone.0166489

4. Kretschmer M, Mamistvalov R, Sprinzak D, Vollmar AM, Zahler S. Matrix stiffness regulates Notch signaling activity in endothelial cells. Journal of Cell Science. 2023 Jan 15;136(2):jcs260442. doi:10.1242/jcs.260442

5. Abedi H, Zachary I. Vascular Endothelial Growth Factor Stimulates Tyrosine Phosphorylation and Recruitment to New Focal Adhesions of Focal Adhesion Kinase and Paxillin in Endothelial Cells*. Journal of Biological Chemistry. 1997 Jun 13;272(24):15442–51. doi:10.1074/jbc.272.24.15442

6. Sun M, Spill F, Zaman MH. A computational model of YAP/TAZ mechanosensing. Vol. 110. 2016;110(11):2540–50.

7. Provenzano PP, Inman DR, Eliceiri KW, Keely PJ. Matrix density-induced mechanoregulation of breast cell phenotype, signaling and gene expression through a FAK–ERK linkage. Oncogene. 2009 Dec;28(49):4326–43. doi:10.1038/onc.2009.299

8. Sako Y, Hibino K, Miyauchi T, Miyamoto Y, Ueda M, Yanagida T. Single-Molecule Imaging of Signaling Molecules in Living Cells. Single Molecules. 2000;1(2):159–63. doi:10.1002/1438-5171(200006)1:2<159::AID-SIMO159>3.0.CO;2-4

9. Holmes WR, Lin B, Levchenko A, Edelstein-Keshet L. Modelling Cell Polarization Driven by Synthetic Spatially Graded Rac Activation. PLOS Computational Biology. 2012 Jun 21;8(6):e1002366. doi:10.1371/journal.pcbi.1002366

10. Dawes AT, Edelstein-Keshet L. Phosphoinositides and Rho proteins spatially regulate actin polymerization to initiate and maintain directed movement in a one-dimensional model of a motile cell. Biophys J. 2007 Feb 1;92(3):744–68. doi:10.1529/biophysj.106.090514 PubMed PMID: 17098793; PubMed Central PMCID: PMC1779977.

11. Feng J, Ito M, Kureishi Y, Ichikawa K, Amano M, Isaka N, et al. Rho-associated Kinase of Chicken Gizzard Smooth Muscle*. Journal of Biological Chemistry. 1999 Feb 5;274(6):3744–52. doi:10.1074/jbc.274.6.3744

12. Ji H, Tang H, Lin H, Mao J, Gao L, Liu J, et al. Rho/Rock cross-talks with transforming growth factor-β/Smad pathway participates in lung fibroblast-myofibroblast differentiation. Biomed Rep. 2014 Nov;2(6):787–92. doi:10.3892/br.2014.323 PubMed PMID: 25279146; PubMed Central PMCID: PMC4179758.

13. Tania N, Prosk E, Condeelis J, Edelstein-Keshet L. A temporal model of cofilin regulation and the early peak of actin barbed ends in invasive tumor cells. Biophys J. 2011 Apr 20;100(8):1883–92. doi:10.1016/j.bpj.2011.02.036 PubMed PMID: 21504724; PubMed Central PMCID: PMC3077689.

14. Song X, Chen X, Yamaguchi H, Mouneimne G, Condeelis JS, Eddy RJ. Initiation of cofilin activity in response to EGF is uncoupled from cofilin phosphorylation and dephosphorylation in carcinoma cells. J Cell Sci. 2006 Jul 15;119(Pt 14):2871–81. doi:10.1242/jcs.03017 PubMed PMID: 16803871.

15. Cirit M, Krajcovic M, Choi CK, Welf ES, Horwitz AF, Haugh JM. Stochastic Model of Integrin-Mediated Signaling and Adhesion Dynamics at the Leading Edges of Migrating Cells. PLOS Computational Biology. 2010 Feb 26;6(2):e1000688. doi:10.1371/journal.pcbi.1000688

16. Yang X, Yu K, Hao Y, Li D ming, Stewart R, Insogna KL, et al. LATS1 tumour suppressor affects cytokinesis by inhibiting LIMK1. Nat Cell Biol. 2004 Jul;6(7):609–17. doi:10.1038/ncb1140

17. Pollard TD. Rate constants for the reactions of ATP- and ADP-actin with the ends of actin filaments. J Cell Biol. 1986 Dec;103(6 Pt 2):2747–54. doi:10.1083/jcb.103.6.2747 PubMed PMID: 3793756; PubMed Central PMCID: PMC2114620.

18. Higashida C, Suetsugu S, Tsuji T, Monypenny J, Narumiya S, Watanabe N. G-actin regulates rapid induction of actin nucleation by mDia1 to restore cellular actin polymers. J Cell Sci. 2008 Oct 15;121(Pt 20):3403–12. doi:10.1242/jcs.030940 PubMed PMID: 18827014.

19. Passier M, Bentley K, Loerakker S, Ristori T. YAP/TAZ drives Notch and angiogenesis mechanoregulation in silico. npj Syst Biol Appl. 2024 Oct 5;10(1):1–16. doi:10.1038/s41540-024-00444-3

20. Matsuo E, Okamoto T, Ito A, Kawamoto E, Asanuma K, Wada K, et al. Substrate stiffness modulates endothelial cell function via the YAP-Dll4-Notch1 pathway. Experimental Cell Research. 2021 Nov;408(1):112835. doi:10.1016/j.yexcr.2021.112835

21. Gabhann FM, Popel AS. Targeting Neuropilin-1 to Inhibit VEGF Signaling in Cancer: Comparison of Therapeutic Approaches. PLOS Computational Biology. 2006 Dec 29;2(12):e180. doi:10.1371/journal.pcbi.0020180

22. Sarabipour S, Kinghorn K, Quigley KM, Kovacs-Kasa A, Annex BH, Bautch VL, et al. Trafficking dynamics of VEGFR1, VEGFR2, and NRP1 in human endothelial cells. PLoS Comput Biol. 2024 Feb 7;20(2):e1011798. doi:10.1371/journal.pcbi.1011798 PubMed PMID: 38324585; PubMed Central PMCID: PMC10878527.

23. Djokovic D, Trindade A, Gigante J, Pinho M, Harris AL, Duarte A. Incomplete Dll4/Notch signaling inhibition promotes functional angiogenesis supporting the growth of skin papillomas. BMC Cancer. 2015 Aug 28;15:608. doi:10.1186/s12885-015-1605-2 PubMed PMID: 26314892; PubMed Central PMCID: PMC4552132.

24. Kühn C, Checa S. Computational Modeling to Quantify the Contributions of VEGFR1, VEGFR2, and Lateral Inhibition in Sprouting Angiogenesis. Front Physiol. 2019 Mar 27;10:288. doi:10.3389/fphys.2019.00288 PubMed PMID: 30971939; PubMed Central PMCID: PMC6445957.

25. Jakobsson L, Franco CA, Bentley K, Collins RT, Ponsioen B, Aspalter IM, et al. Endothelial cells dynamically compete for the tip cell position during angiogenic sprouting. Nat Cell Biol. 2010 Oct;12(10):943–53. doi:10.1038/ncb2103
