# Supplementary material for: Targeting stiffness-dependent YAP/TAZ restores angiogenesis dynamics impaired by ALK1 knockout in silico
Source: PLoS Comput Biol. 2026 Jul 16;22(7):e1013561. doi: 10.1371/journal.pcbi.1013561 (PMC13387619; doi:10.1371/journal.pcbi.1013561)
Supplement: S2 Table — (DOCX) [file pcbi.1013561.s006.docx]

**S2 Table**

Upper and lower boundaries for parameter fits

| Parameter | Lower boundary | Upper boundary |
| --- | --- | --- |
| $C_{\varphi}$ | 7.5 | 50 |
| $n_{2}$ | 0.01 | 4 |
| $k_{sfdf}$ | 0.0675 | 6.75 |

These boundaries were determined based on the previous study by Sun et al.(1), where we used approximately 20 kPa above and below the original average for $C_{\varphi}$ as its boundaries, 10 times higher and lower than the original for $k_{sfdf}$ and similar boundaries to a previous fitting for $n_{2}$ (2).

**References**

1. Sun M, Spill F, Zaman MH. A computational model of YAP/TAZ mechanosensing. Vol. 110. 2016;110(11):2540–50.

2. Passier M, Bentley K, Loerakker S, Ristori T. YAP/TAZ drives Notch and angiogenesis mechanoregulation in silico. npj Syst Biol Appl. 2024 Oct 5;10(1):1–16. doi:10.1038/s41540-024-00444-3
